# Frustrated Lewis Pairs and Metallic Ni Synergistically Enabled Low‐Temperature Hydrogenolysis of Lignin Models

**DOI:** 10.1002/advs.202520585

**Published:** 2026-05-25

**Authors:** Jinpeng Liang, Ziyi Ma, Zhaoxi Cai, Kongqian Liang, Chenglei Xiao, Sitong Chen, Na Wang, Zhimin Xue, Chao Xie, Jinliang Song

**Affiliations:** ^1^ School of Chemical Engineering and Light Industry Guangdong University of Technology Guangzhou China; ^2^ State Key Laboratory of Efficient Production of Forest Resources College of Materials Science and Technology Beijing Forestry University Beijing China; ^3^ National & Local Joint Engineering Research Center on Biomass Resource Utilization College of Environmental Science and Engineering Nankai University Tianjin China

**Keywords:** C‐O cleavage, frustrated Lewis pairs, lignin valorization, low temperature hydrogenolysis, non‐noble metals

## Abstract

Low‐temperature hydrogenolysis of aromatic ether bonds is a highly promising strategy to convert lignin. However, it remains highly challenging to enable this strategy over non‐noble metal‐based catalysts. Herein, Ni and Al mixed oxide catalysts (denoted as NiAlO_x_‐T, where T represented the reduction temperature) were constructed by the reduction of layered Ni‐Al hydroxides. Interestingly, the NiAlO_x_‐300 could efficiently promote the hydrogenolysis of various lignin models (i.e., α‐O‐4 referring to benzyl phenyl ether linkage (C_α_‐O‐Ar), β‐O‐4 referring to 2‐phenoxy‐1‐phenylethanol linkage (C_β_‐O‐Ar), and 4‐O‐5 referring to diphenyl ether linkage (Ar‐O‐Ar)) at a temperature of ≤ 100°C. Notably, benzyl phenyl ether (a typical α‐O‐4 model) could be converted into toluene and phenol at 20°C. Systematic analysis revealed that abundant solid surface frustrated Lewis pairs (ssFLPs) composed by unsaturated Al sites (Al^3+^_unsatur._) proximal to a surface oxygen vacancy and surface Lewis basic OH were generated in NiAlO_x_‐300. The excellent performance of the NiAlO_x_‐300 catalyst originated from the synergistic effect of ssFLPs sites and metallic Ni^0^ sites, which was beneficial to efficient hydrogen activation for hydrogenolysis of lignin models at mild conditions. This work provided a new kind of non‐noble metal‐based catalyst for lignin valorization by creating solid surface frustrated Lewis pairs cooperated with non‐noble metals.

## Graphical abstract

Nickel–aluminum composite oxide (NiAlO_x_‐300) derived from NiAl layered double hydroxide showed high activity for hydrogenolysis of various lignin models. Especially, hydrogenolysis of benzyl phenyl ether could be efficiently proceeded at 20°C and 1 MPa H_2_. Systemic study revealed that ssFLPs sites and metallic Ni^0^ synergistically resulted in the excellent performance of NiAlO_x_‐300 on hydrogenolysis of lignin models at mild reaction temperatures and 1 MPa H_2_.

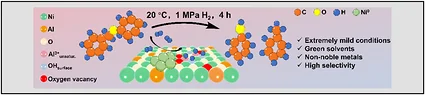

## 1 Introduction

Lignin has been recognized as a renewable and abundantly available aromatic carbon resource to produce value‐added chemicals, sustainable fuels, and functional materials [1, 2, 3, 4, 5]. Structurally, lignin is a complex, highly branched, and 3D polyphenolic substance containing plenty of aromatic ether (C─O) and C─C linkages [6, 7, 8, 9, 10]. Especially, ca. 70% of the structural units in lignin are linked through aromatic C─O ether bonds. Thereby, the cleavage of the aromatic C─O bonds has become the core for the upgrading of lignin and its derivatives/models [9].

Of the proposed strategies to cleave the aromatic C─O bonds, hydrogenolysis shows great potential in practical applications owing to its atom‐economic and environmentally benign characteristics. In recent years, substantial noble metal‐based catalytic systems, e.g., Pd [11, 12, 13, 14, 15], Ru [16, 17, 18, 19, 20, 21, 22], Pt [23, 24, 25], and Rh [26, 27], have been developed for hydrogenolysis of aromatic C─O bond in lignin and its models. Despite their good catalytic performance, the major obstacle for noble metals is their high cost, which significantly limits their large‐scale applications. In this context, non‐noble metals (especially Ni and Co) with the ability to cleave the aromatic C─O bond via hydrogenolysis attract tremendous interest, and several Ni and Co‐based catalysts have been explored. For example, the Co/C@N catalyst exhibited good activity on hydrogenolysis of benzyl phenyl ether (a α‐O‐4 lignin model) at 160°C and a H_2_ pressure of 2 MPa [28]. Besides, N‐doped carbon‐supported Ni‐based catalyst (Ni@NC‐800) could promote the complete hydrogenolysis of 2‐phenylethyl phenyl ether (a β‐O‐4 lignin model) at 200°C and a H_2_ pressure of 1 MPa [29]. Despite these achievements, most of the developed catalytic systems still suffered from high temperature and high H_2_ pressure [30, 31, 32]. Recently, Jiang et al. developed a Ni/AlP_0.5_O_x_ catalyst for hydrogenolysis of lignin‐derived aryl ethers [33]. Although this catalyst could catalyze hydrogenolysis of α‐O‐4 models at 30°C or 50°C, a high temperature (150°C) was needed for hydrogenolysis of β‐O‐4 and 4‐O‐5 models. To date, it is still highly desirable for robust non‐noble metal‐based catalysts with broad substrate adaptability for hydrogenolysis of lignin models at mild reaction temperatures (e.g., below 100°C).

To enable hydrogenolysis of lignin models to occur efficiently, the core point is effective activation of H_2_. As well recognized, H_2_ can be effectively activated by frustrated Lewis pairs (FLPs) in homogeneous molecular‐based complexes and some heterogeneous catalysts [34, 35, 36]. Especially, solid surface FLPs (ssFLPs) can be easily constructed by the generation of surface oxygen vacancies or hydroxyl‐metal ion pairs on some transition metal (e.g., Ni, Ce, In, and Al) oxides [37, 38, 39, 40]. These metal oxide‐derived catalysts with appropriate ssFLPs have been proven to display good ability to activate H_2_ for hydrogenation reactions. However, the feasibility of promoting hydrogenolysis of lignin models by ssFLPs is still unidentified. Besides H_2_ activation, the activation of the aromatic C─O ether bonds is another parameter that affects the reactivity of hydrogenolysis of lignin models. Generally, the aromatic C─O ether bonds can be activated by the interaction between the ether bonds and the acidic sites on catalysts [16]. In the catalytic materials with abundant ssFLPs, the Lewis acidic sites in ssFLPs can play the role of activating C─O ether bonds, thus promoting hydrogenolysis of lignin models. Recently, Yu et al. reported that hydrogenolysis of the C─O bond in lignin models could be efficiently facilitated by the synergistic effect of Ni^0^ and Lewis acidic sites in a Ni/C@N catalyst [41], indicating that the cooperation between metal sites and Lewis sites would be a feasible approach to boost hydrogenolysis of lignin models. Therefore, inspired by the unique features of ssFLPs in activating H_2_ and C─O ether bonds, the fabrication of non‐noble metal catalysts with effective ssFLPs is greatly promising to drive mild‐temperature hydrogenolysis of lignin models. Nonetheless, rational and controllable fabrication of non‐noble metal catalysts with appropriate ssFLPs remains a great challenge for mild‐temperature hydrogenolysis of various lignin models, including α‐O‐4, β‐O‐4, and 4‐O‐5 linages.

Layered double hydroxides (LDHs) have garnered considerable interest in the field of heterogeneous catalysis [42, 43, 44]. LDHs can act as precursors to enable continuous tuning of catalyst components, defects, hybridizations, and topological structures [45, 46, 47]. Especially, LDHs can be constructed by coprecipitation of reducible metals (e.g., Co and Ni) and Al^3+^ salts. As reported, ssFLPs can be effectively generated on reducible metal oxides and non‐reducible aluminum hydroxide [41, 48]. Thus, abundant ssFLPs could be promisingly produced by rational treatment of NiAl‐LDHs or CoAl‐LDHs, which is beneficial to fabricating a highly potential catalyst for mild‐temperature hydrogenolysis of lignin models.

Inspired by the unique properties of LDHs materials, in this work, NiAlO_x_‐T materials (in which T was the reduction temperature) were fabricated by reduction of the NiAl‐LDH precursor in the H_2_ atmosphere. The synthesized NiAlO_x_‐300 was observed to possess excellent catalytic activity on hydrogenolysis of various lignin models (e.g., α‐O‐4, β‐O‐4, and 4‐O‐5) at mild reaction temperatures of below 100°C (even 20°C for benzyl phenyl ether, a typical α‐O‐4 model), resulting from the synergistic effect of ssFLPs (Al^3+^_unsatur_ (unsaturated Al site)‐OH_surface_ (surface Lewis basic OH) and the metallic Ni^0^ sites. The fact that NiAlO_x_‐300 displayed wide applicability to diverse lignin models at below 100°C represented a breakthrough in this area, confirming its great potential.

## 2 Results and Discussion

### 2.1 Characterization of the Fabricated NiAlO_x_‐T Catalytic Materials

**FIGURE 1:**
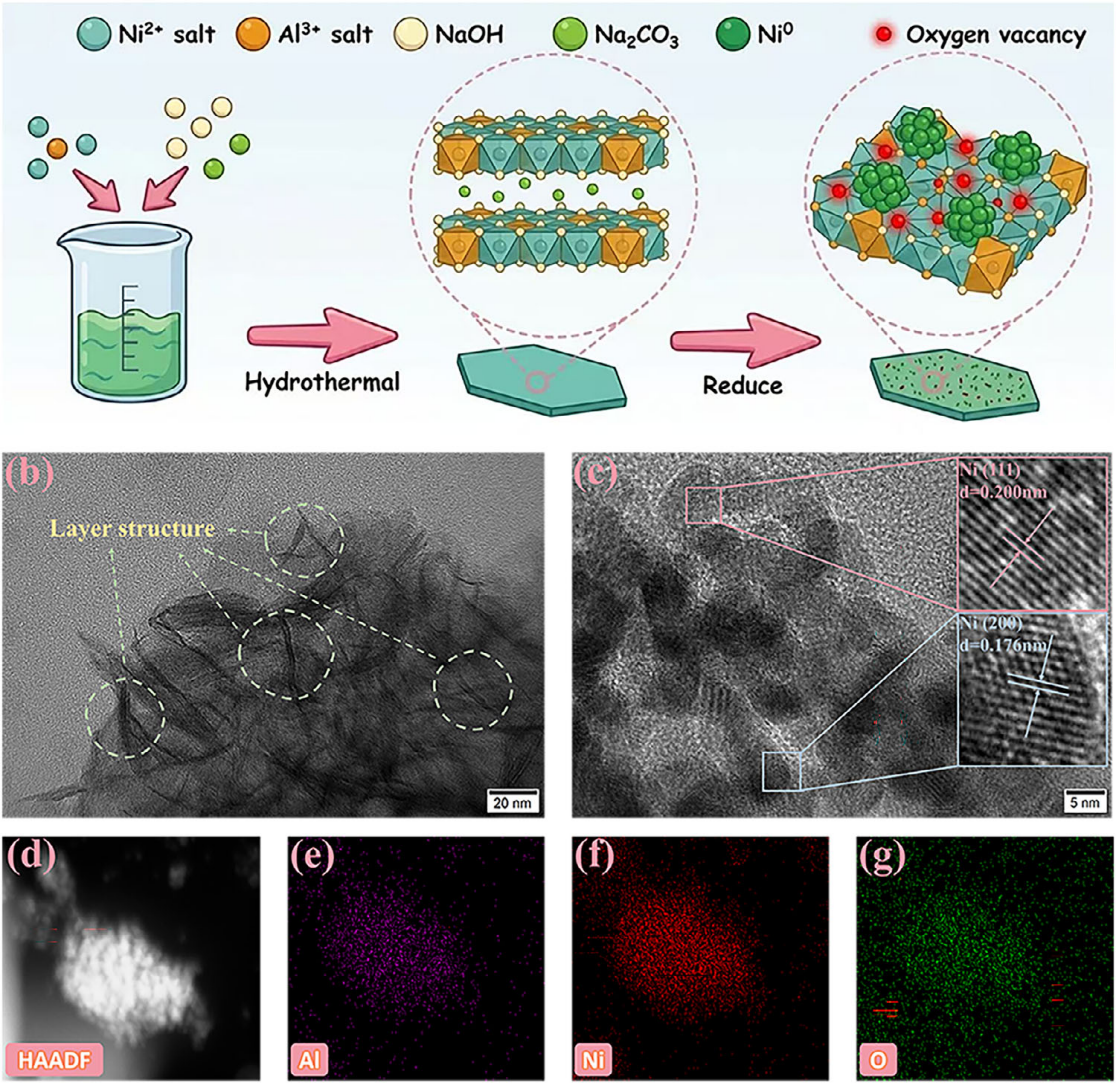
Synthesis and morphological characterization of the NiAlO_x_‐T materials. (a) Schematic diagram of the synthetic process of NiAlO_x_‐T, (b) TEM image of NiAl‐LDH, (c) HRTEM image of NiAlO_x_‐300, and (d–g) EDS element mappings of the NiAlO_x_‐300 sample via HAADF‐STEM.

**FIGURE 2:**
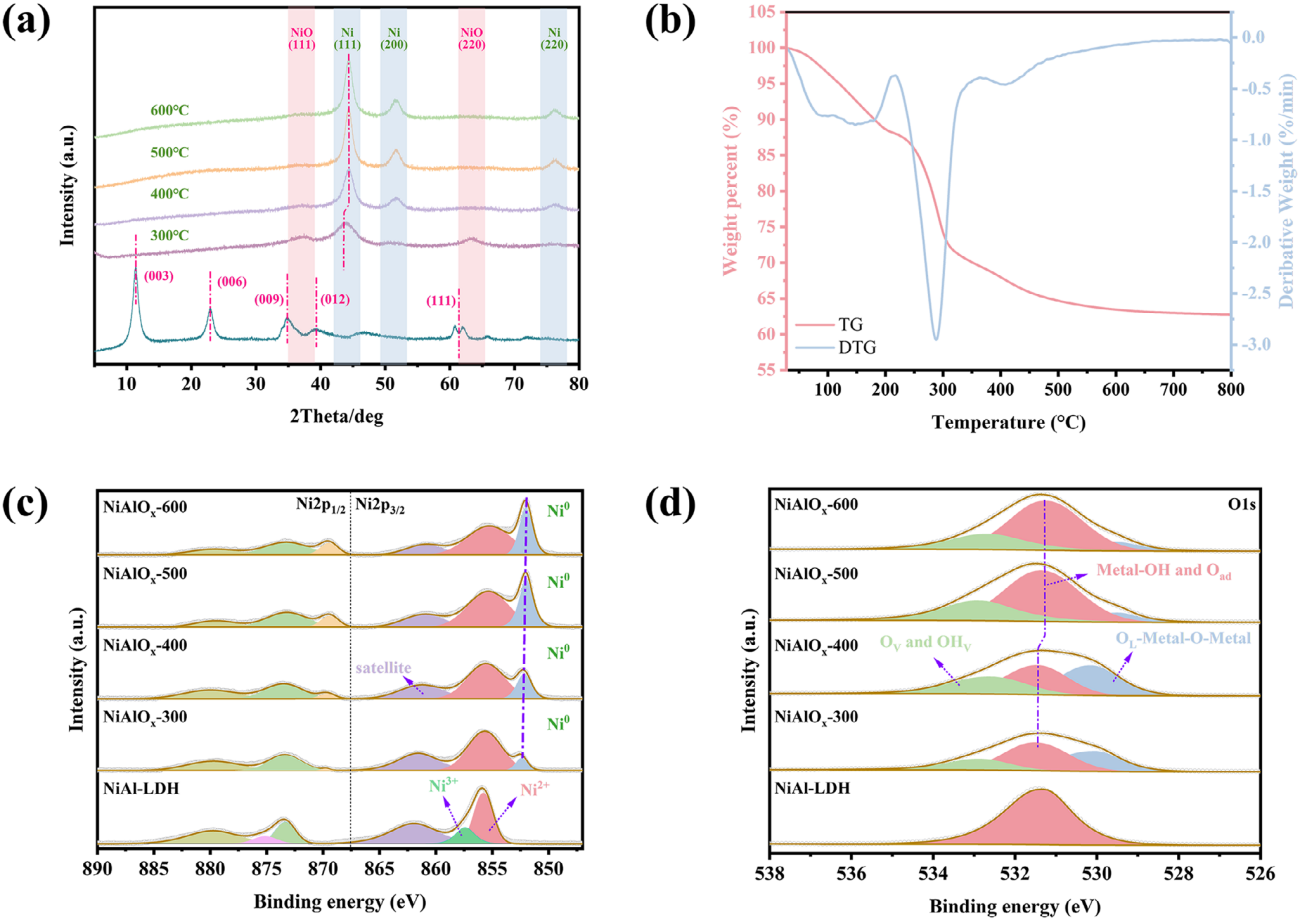
Characterization of the NiAlO_x_‐T materials. (a) XRD patterns, (b) TGA curve of NiAl‐LDH, (c) XPS spectra of Ni 2p, and (d) XPS spectra of O 1s.

To obtain the desired NiAlO_x_‐T catalysts, the NiAl‐LDH precursor was reduced at different temperatures (i.e., 300°C, 400°C, 500°C, and 600°C) to control the structural transformation of NiAl‐LDH (Figure 1a). The detailed synthetic process was comprehensively described in the Experimental Section of the Supporting Information. To investigate the decomposition of NiAl‐LDH precursor in the reduction process, thermogravimetry (TG) examination under N_2_ atmosphere was performed (Figure 2b). The initial mass decrease observed from 30°C to 200°C was primarily caused by the elimination of both surface‐bound and interlamellar water molecules [49]. The pronounced mass decrease at ca. 280°C was associated with the collapse of hydroxyl groups and the liberation of carbonate species as gaseous CO_2_. After the heating temperature was beyond 400°C, no significant decrease was observed in the residual mass, signifying that the major decomposition process, i.e., transformation of NiAl‐LDH precursor into NiAlO_x_ phases, was basically completed.

The morphological transformation could be confirmed via TEM examinations. Obviously, the NiAl‐LDH precursor displayed a typical 2D nanosheet structure with smooth surfaces (Figure 1b and Figure S1). In comparison, the synthesized NiAlO_x_‐T materials showed the nanoflake‐like structure after the reduction treatment, and Ni nanoparticles were uniformly dispersed on the NiAlO_x_ samples (Figure S2). The size of Ni nanoparticles grew gradually from 3.31 to 8.65 nm when the reduction temperature increased from 300°C to 600°C. Nevertheless, there was no significant agglomeration because the generated CO_2_ during the thermal treatment process could act as a physical barrier to prevent particle coalescence in metal oxide systems [50]. Moreover, there were characteristic lattice fringes at the interplanar distance of 0.176 and 0.200 nm in the TEM image of NiAlO_x_‐T materials (Figure 1c), which were assigned to (200) plane and the (111) plane of metallic Ni^0^ [51], further confirming the formation of Ni^0^ in the NiAlO_x_‐T materials (taking NiAlO_x_‐300 as an example). Besides, EDS elemental mapping suggested the homogeneous spatial distribution of all constituent elements with metallic nickel, exhibiting no detectable agglomeration phenomena (Figure 1d–g). Additionally, based on the results from N_2_ adsorption–desorption examination (Table 1 and Figure S3), all the prepared NiAlO_x_‐T catalysts displayed type IV isotherms (Figure S3a) [52], indicating the existence of plenty of mesopores in the four NiAlO_x_‐T materials with a wide distribution in the mesopore size (Figure S3b). Meanwhile, as the reduction temperature increased, the specific surface area of the prepared NiAlO_x_‐T underwent a significant decrease (from 200.9 to 115.6 m^2^·g^−1^). Notably, when the reduction temperature reached 600°C, the pore volume of the NiAlO_x_‐600 significantly decreased to 0.35 cm^3^·g^−1^, while that of the other three materials was around 0.45 cm^3^·g^−1^ (Table S1). These results meant the collapse of the pore channels at higher reduction temperature (e.g., 600°C).

**TABLE 1:** Catalytic performance of various catalysts on hydrogenolysis of benzyl phenyl ether.^[a]^

			Yield (%)^[b]^			
Entry	Catalyst	Conversion (%)^[b]^	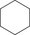	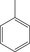	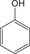	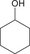
1	NiAl‐LDH	0	0	0	0	0
2	NiAlO_x_‐300	81.1	0	41.2	39.9	trace
3	NiAlO_x_‐400	75.3	0	38.7	36.6	trace
4	NiAlO_x_‐500	74.9	0	38.6	36.4	trace
5	NiAlO_x_‐600	35.8	0	18.6	17.2	trace
6^[c]^	Ni/NiO	0	0	0	0	0
7^[d]^	AlO_x_(OH)_y_‐300	0	0	0	0	0
8^[e]^	NiAlO_x_‐300	0	0	0	0	0
9^[f]^	NiAlO_x_‐300	31.7	0	16.4	15.3	trace
10^[g]^	NiAlO_x_‐300	0	0	0	0	0
11^[h]^	NiAlO_x_‐300	0	0	0	0	0

^[a]^ Reaction conditions: Benzyl phenyl ether, 1.0 mmol; ethanol, 5 g; reaction temperature, 30°C; reaction time, 1 h; 1 MPa H_2_; amount of catalyst, 45 mg.

^[b]^ Conversion and yield were determined by GC using *n*‐dodecane as the internal standard.

^[c]^ 45 mg, and it was obtained from the reduction of Ni(OH)_2_ at 300°C.

^[d]^ 45 mg, and it was obtained from the reduction of AlOOH at 300°C.

^[e]^ 1 MPa N_2_, 30°C.

^[f]^ 1 MPa N_2_, 150°C.

^[g]^ 5 × 10^−3^ mol·L^−1^ pyridine was used as additive.

^[h]^ 5 × 10^−3^ mol·L^−1^ benzoic acid was used as an additive.

The fine structure of NiAlO_x_‐T materials was further examined by X‐ray diffraction (XRD) and X‐ray photoelectron spectroscopy (XPS) techniques. XRD pattern of the NiAl‐LDH precursor exhibited the characteristic reflections of LDH materials (PDF#00‐051‐0045) with seven reflection peaks centered at ca. 11.4°, 23.1°, 34.1°, 38.7°, 45.8°, 59.8°, and 61.1° (Figure 2a) [53]. In comparison, in the XRD patterns of the synthesized NiAlO_x_‐T, several new peaks appeared at ca. 37.3°, 44.8°, 51.9°, 63.2°, and 76.5°, while all the basal reflections of NiAl‐LDH disappeared, indicating the structural transformation of NiAl‐LDH after the reduction treatment. Among the newly generated peaks, the peaks at ca. 37.3° and 63.2° could be assigned to (111) and (220) planes of the NiO [50], while the other three peaks at ca. 44.8°, 51.9°, and 76.5° originated from (111), (200), and (220) planes of metallic Ni [54]. Besides, no reflection peaks of Al_2_O_3_ in the XRD patterns of NiAlO_x_‐T were detected, indicating the amorphous property of the generated AlO_x_ species. Especially, with the increase of reduction temperature from 300°C to 600°C, the peak intensity of metallic Ni enhanced gradually while that of the NiO‐like phase showed a reverse tendency and almost disappeared at 400°C, suggesting a gradual reduction of NiO‐like phase to metallic Ni with the increased reduction temperature. Moreover, the chemical composition of the NiAlO_x_‐T materials was determined by the XPS technique (Figure 2 and Figure S4). High‐resolution XPS spectra of Ni 2p in the NiAl‐LDH precursor (Figure 2c) showed that there were three Ni species with the corresponding binding energies at ca. 855.8, 857.5, and 862 eV (Ni 2p^3/2^ region), which were attributed to Ni^2+^, Ni^3+^, and Ni^2+^ satellite peaks, respectively [55, 56, 57]. After the formation of NiAlO_x_‐T materials, the Ni^3+^ peak disappeared, and a new peak was observed at the binding energy of ca. 852.5 eV, which was assigned to metallic Ni^0^, confirming the reduction of Ni^2+^/Ni^3+^ species into metallic Ni. More importantly, the surface content of Ni^0^ increased progressively as the reduction temperature increased, whereas the surface content of Ni^2+^ decreased. Interestingly, a small amount of metallic Ni was found in the NiAlO_x_‐300, indicating that the Ni^2+^/Ni^3+^ species in NiAl‐LDH were easily reduced by H_2_ into metallic Ni under relatively mild temperature (300°C). The signal of O 1s in the four NiAlO_x_‐T materials could be divided into three peaks at ca. 529.1–529.8, 531.1–531.4, and 532.6–532.8 eV (Figure 2d), which corresponded to oxygen in lattice structure (O_L_), hydroxyl bound with Ni and Al (Al─OH and Ni─OH) or oxygen species adsorbed (O_ad_) on the surface, and oxygen vacancies (O_V_)/OH defect (OH_V_), respectively [41, 58, 59]. In contrast, the XPS spectra of O 1s in the NiAl‐LDH only showed one peak at 531.4 eV, assigned to the M─OH [41, 58, 59]. For Al 2p in NiAl‐LDH, two peaks at 68.1 and 70.4 eV, which were attributed to Al 2p_3/2_ and Al 2p_1/2_ levels of Al^3+^, could be observed (Figure S4). Besides, another peak at 74.0 eV was assigned to the Al─O bond in Al(OH)_3_ [60, 61]. In contrast to NiAl‐LDH, the XPS peak of Al 2p in the NiAlO_x_‐T materials beyond 67.8 eV could be assigned to unsaturated Al^3+^ species induced by surface hydroxyl defects and oxygen vacancies [41]. As the reduction temperature increased, the binding energy of this peak gradually shifted to lower values with a progressive decrease in its intensity, concomitantly accompanied by an enhancement in the intensity of Al─O bonds (Figure S4). These observations indicated the strengthened interfacial interactions between amorphous alumina and NiO species. To confirm the interaction between NiO‐like phase and amorphous Al_2_O_3_ species, H_2_‐TPR experiments were performed (Figure S5). The TPR profile of NiAl‐LDH exhibited two hydrogen consumption peaks: the one at ca. 150°C was assigned to the reduction of unstable Ni^+^ in the lamellar hydroxide structure, and the main peak centered at 500°C originated from the reduction of a highly dispersed NiO‐like phase interacting with Al_2_O_3_ strongly. It was observed that the main peak in TPR profiles of NiAlO_x_‐T materials shifted toward higher temperature from NiAlO_x_‐300 to NiAlO_x_‐600, and the total integrated area of the reduction peak (H_2_ consumption) decreased gradually, indicating an enhanced interaction between NiO‐like phase and Al_2_O_3_ species, which stabilized the remained NiO‐like phase [54, 62].

### 2.2 Catalyst Evaluation for Hydrogenolysis of Benzyl Phenyl Ether

The catalytic activity of different catalysts was initially evaluated by employing hydrogenolysis of benzyl phenyl ether (a typical α‐O‐4 model compound) as the model reaction (Table 1). Hydrogenolysis of benzyl phenyl ether did not occur using NiAl‐LDH precursor as the catalyst (Table 1, entry 1), suggesting that the unreduced NiAl‐LDH was inert to the reaction. To our surprise, NiAlO_x_‐300 could effectively catalyze the cleavage of the C─O bond in benzyl phenyl ether at 30°C (Table 1, entry 2). The conversion of benzyl phenyl ether reached 81.1% with a reaction time of 1 h, and the products were phenol and toluene. In comparison, no product was observed in the presence of MgAlO_x_‐300, CoAlO_x_‐300, and CuAlO_x_‐400 at the same reaction conditions (Table S2, entries 1–3), suggesting the poor catalytic activity of Mg, Co, and Cu sites on the cleavage of benzyl phenyl ether at 30°C and 1 MPa of H_2_ in EtOH. Moreover, the reduction temperature to prepare NiAlO_x_‐T showed an obvious impact on their catalytic activity. With the increase of the reduction temperature, the catalytic activity of NiAlO_x_‐T catalysts decreased (Table 1, entries 2–5). Especially, the catalytic activity of NiAlO_x_‐600 exhibited a notable decrease (to only 35.8% conversion), although it possessed the highest nickel loading. This decline was attributed to the significant decrease in both acid and base sites on NiAlO_x_‐600 (Table S3) and the agglomeration Ni particles (Figure S2) by the excessively high reduction temperature. Besides, when Ni/NiO (from the reduction of Ni(OH)_2_ at 300°C) was used as the catalyst, no reaction occurred (Table 1, entry 6), confirming the indispensability of AlO_x_ species. In addition, benzyl phenyl ether could be converted under N_2_ atmosphere at 150°C over NiAlO_x_‐300 (Table 1, entries 8 and 9), suggesting that NiAlO_x_‐300 could catalyze hydrogenolysis of benzyl phenyl ether using ethanol as the hydrogen source. This result further confirmed the superior activity of NiAlO_x_‐300 for the hydrogenolysis of benzyl phenyl ether. More importantly, NiAlO_x_‐300 showed much milder reaction conditions for hydrogenolysis of benzyl phenyl ether than most of the reported typical catalysts (Table S4), which was a major breakthrough of this developed NiAlO_x_‐300 catalytic system. Based on the results above, NiAlO_x_‐300 was the optimal catalyst for our catalytic system.

### 2.3 Optimization of Reaction Conditions for Hydrogenolysis of Benzyl Phenyl Ether

Based on the results in Table 1, NiAlO_x_‐300, NiAlO_x_‐400, and NiAlO_x_‐500 showed no statistically significant differences in catalytic activity. Considering the energy efficiency and the principles of green chemistry, the NiAlO_x_‐300 catalyst was selected as the optimal catalyst for subsequent catalytic investigations. Initially, the solvent effect on the hydrogenolysis of benzyl phenyl ether was investigated. To our delight, excellent results could be achieved in various alcohols (Table S5, entries 1–5), including methanol, ethanol, 1‐propanol, isopropanol, and 1‐butanol. The benzyl phenyl ether was not completely converted in 1‐butanol (Table S5, entry 5) owing to the relatively higher viscosity of 1‐butanol at a reaction temperature of 30°C. Besides, *n*‐hexane, as an aprotic apolar solvent, displayed poor performance for the reaction (Table S5, entry 6) probably because the low polarity of *n*‐hexane decreased the interaction between the benzyl phenyl ether (dissolved in *n*‐hexane) and the NiAlO_x_‐300 with a polar surface. Additionally, no reaction was detected when water was used as the reaction solvent (Table S5, entry 7) because of the low solubility of benzyl phenyl ether in water at 30°C. To further confirm the role of alcohols, more discussion on the solvent effect was performed. Because no reaction occurred when the reaction was performed in N_2_ atmosphere at 30°C (Table 1, entry 8), the possibility that the alcohols could act as hydrogen donors could be excluded. Furthermore, a control experiment using a mixed solvent composed of 90% n‑hexane and 10% ethanol was performed (Table S5, entry 8). It was observed that the catalytic activity in the mixed solvent significantly improved in comparison to that in pure n‑hexane (Table S5, entry 6). Considering that ethanol did not play the role of a hydrogen donor, the addition of ethanol into n‑hexane could probably improve the polarity of the mixed solution, enhancing the interaction between the substrate and the catalyst, thereby markedly boosting the catalytic activity. Based on the properties of the studied solvent, ethanol was selected as the final reaction solvent.

**FIGURE 3:**
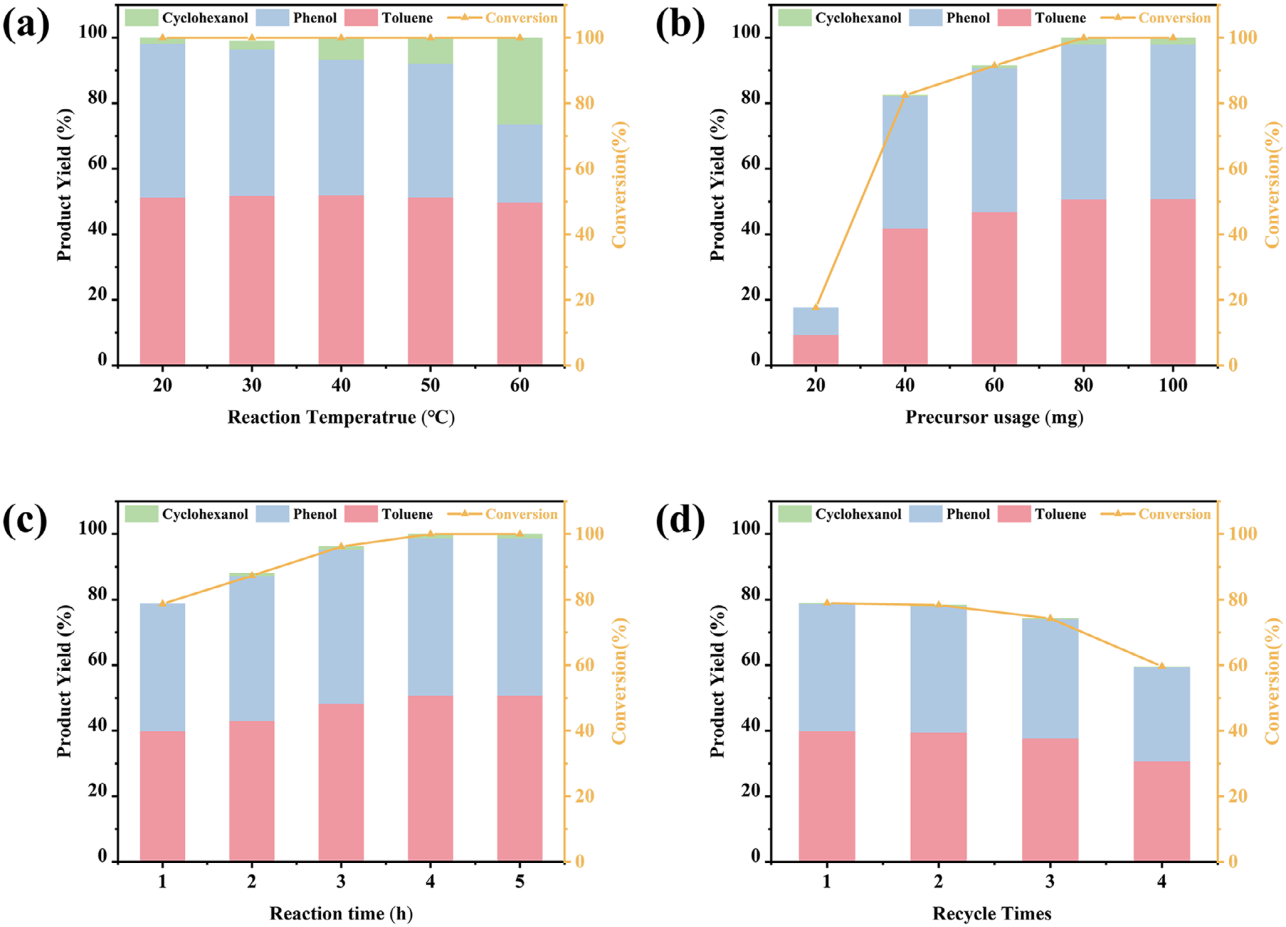
Optimization of reaction parameters. (a) Effect of reaction temperature. Reaction conditions: benzyl phenyl ether, 1 mmol; EtOH, 5 g; NiAlO_x_‐300, 45 mg; reaction time, 4 h; 1 MPa H_2_. (b) Influence of catalyst precursor usage. Reaction conditions: benzyl phenyl ether, 1 mmol; reaction temperature, 30°C; EtOH, 5 g; reaction time, 4 h; 1 MPa H_2_. (c) Effect of reaction time. Reaction conditions: benzyl phenyl ether, 1 mmol; EtOH, 5 g; NiAlO_x_‐300, 45 mg; reaction temperature, 30°C; 1 MPa H_2_. (d) Reusability of the NiAlO_x_‐300 catalyst. Reaction conditions: benzyl phenyl ether, 1 mmol; reaction temperature, 30°C; EtOH, 5 g; NiAlO_x_‐300, 45 mg; reaction time, 1 h; 1 MPa H_2_.

Generally, reaction temperature could significantly affect the catalytic performance of a catalyst. As shown in Figure 3a, benzyl phenyl ether could be completely converted in the studied temperature range (20°C–60°C) with a reaction time of 4 h. Especially, the C─O bond in benzyl phenyl ether was completely hydrogenlyzed over NiAlO_x_‐300 at 20°C, which represented the mildest temperature for this transformation (Table S4, entry 10). Importantly, reaction temperature had an obvious impact on product selectivity. When the reaction was conducted at 20°C, the predominant products were toluene and phenol with a very small amount of cyclohexanol (1.7% yield). With the increase of reaction temperature, more cyclohexanol was generated, and its yield could reach 26.3% at 60°C. This result indicated that high temperature was beneficial for further hydrogenation of the phenol. Considering the practicality and convenience for operation, the optimal temperature was determined to be 30°C. Moreover, the effect of the usage of NiAlO_x_‐300 precursor (NiAl‐LDH) on reaction efficiency was detected (Figure 3b). The conversion of benzyl phenyl ether increased from 17.6% to 100% when the amount of Ni/AlO_x_‐300 precursor increased from 20 to 100 mg, which was caused by the fact that more active sites were accessible to benzyl phenyl ether with a higher catalyst loading. Meanwhile, phenol and toluene were the major products across all tested catalyst loadings, although very few cyclohexanol were detected with a higher catalyst usage. Based on the results in Figure 3b, 80 mg (ca. 45 mg NiAlO_x_‐300) was employed as the optimal catalyst precursor usage. Besides, the time‐dependent kinetic behavior of benzyl phenyl ether conversion was systematically investigated over a reaction period of 1–5 h (Figure 3c), and it was observed that the reaction could be completed with a reaction time of 4 h at 30°C. Notably, benzyl phenyl ether achieved a conversion of 78.2% within 1 h, demonstrating the catalyst's exceptional capability in rapid substrate activation.

Reusability was one of the important advantages of heterogeneous catalysts. Herein, the stability of NiAlO_x_‐300 was tested under the optimized conditions (Figure 3d). Clearly, NiAlO_x_‐300 could be reused for at least three catalytic runs without an obvious decrease in conversion of benzyl phenyl ether and product distribution. As characterized by XRD and TEM (Figures S6 and S7), no obvious change was observed between the fresh NiAlO_x_‐300 and the recovered catalyst, which likewise confirmed the stability of the NiAlO_x_‐300 catalyst. The decreased catalytic activity of NiAlO_x_‐300 in the fourth cycle was probably caused by the slight oxidation of the catalyst surface. Therefore, the spent NiAlO_x_‐300 (after four cycles) was characterized by XRD (Figure S6), TEM (Figure S7), and CO_2_/NH_3_‐TPD (Table S3). As shown in the XRD pattern (Figure S6), a weak but discernible diffraction peak of NiO appeared in the spent NiAlO_x_‐300, while the metallic Ni^0^ peaks became slightly broader and less intense. Meanwhile, TEM analysis (Figure S7) revealed that the Ni nanoparticles in the spent catalyst remain well dispersed with an average particle size of 3.4 nm, which was very similar to that of the fresh catalyst (3.3 nm). Furthermore, there was no obvious change on the basic and acidic sites after four catalytic cycles based on the CO_2_/NH_3_‐TPD results (Table S3). These combined observations indicated that the slight deactivation after three cycles was caused by partial surface oxidation of Ni^0^ species rather than the sintering of Ni^0^ particles and the obvious change of Lewis pairs. Additionally, the decreased catalytic activity of NiAlO_x_‐300 in the fourth cycle was to some extent caused by the magnetism, which caused NiAlO_x_‐300 be adsorbed by the magnetic stirring bar, thus resulting in the mass loss of NiAlO_x_‐300 in the recycled process. After the amount of NiAlO_x_‐300 was supplemented to 45 mg (ca. 5 mg new catalyst), the catalytic activity would be restored. Although the catalytic activity decreased after four catalytic cycles, NiAlO_x_‐300 still retained over 80% of its initial activity (Figure 3d), demonstrating its better recyclability among the reported non‑noble Ni‑based catalysts under such mild conditions (30°C, and 1 MPa H_2_). More importantly, conversion of benzyl phenyl ether and product distribution plateaued after NiAlO_x_‐300 was removed from the reaction system after 1 h (Figure S8), Besides, the concentration of Ni and Al in the reaction mixture was negligible based on ICP‐AES results. These results indicated the heterogeneous nature of NiAlO_x_‐300 in the catalytic process.

### 2.4 Hydrogenolysis of Different Lignin Models

Inspired by the excellent catalytic activity for hydrogenolysis of benzyl phenyl ether, the adaptation of the NiAlO_x_‐300 catalyst in catalytic hydrogenolysis of other lignin models containing different C─O linkages (e.g., α‐O‐4, β‐O‐4, 4‐O‐5, and aryl‐O‐CH_3_) was further demonstrated (Table 2). It was observed that all the examined α‐O‐4 models could be completely converted at 50°C and 1 MPa H_2_ with a reaction time of 5 h (Table 2, entries 1–4), selectively yielding toluene and phenols/cyclohexanediol. Owing to the high hydrogenation reactivity of the generated 1,4‐dihydroxybenzene from 4‐benzyloxyphenol, the major products from this model compound were toluene and cyclohexanediol (Table 2, entry 4), while toluene and the corresponding phenols were the main products from the other three α‐O‐4 models (Table 2, entries 1–3). In comparison, β‐O‐4 and 4‐O‐5 models possessed higher bond dissociation energies than α‐O‐4 models (Table S6). Therefore, higher temperatures (80°C–100°C) were required to convert β‐O‐4 and 4‐O‐5 models over NiAlO_x_‐300. For all the investigated 4‐O‐5 substrates (Table 2, entries 5–8), a reaction temperature of 100°C was necessary to make the reaction complete in 5 or 7 h. Meanwhile, the major products from 4‐O‐5 substrates were cyclohexane and cyclohexanol derivatives because the relatively high temperature (100°C) promoted hydrogenation of the benzene ring. For β‐O‐4 models (Table 2, entries 9 and 10), the major products were ethylbenzene, cyclohexanol, and other derivatives at 80°C (for phenoxyethylbenzene of entry 9) or 100°C (2‐phenoxyacetophenone of entry 10). Besides, the NiAlO_x_‐300 catalyst could also catalyze hydrogenolysis of guaiacol (a typical lignin‐derivative containing aryl─O─CH_3_ bond) at 120°C (Table 2, entry 11). Notably, the cleavage of the aromatic C─O bonds in β‐O‐4 models, 4‐O‐5 models, and guaiacol diminished at the reaction conditions (80°C–120°C), attributed to competitive pathways between the hydrogenolysis of C─O bonds and the direct hydrogenation of aromatic rings. Elevated temperatures simultaneously promoted C─O scission and ring saturation, generating cycloalkane and cyclohexanol via a cascade reaction process. To better assess the adaptability of the NiAlO_x_‐300 catalyst, three lignin models (2,6‐dimethoxyphenol, 3‐benzyloxy‐4‐methoxybenzaldehyde, and 2‐methoxydiphenylether) with methoxy groups were investigated (Table 2, entries 12–14). Gratifyingly, all these three models could be completely converted over NiAlO_x_‐300 at 50°C for 3‐benzyloxy‐4‐methoxybenzaldehyde or 150°C for 2,6‐dimethoxyphenol, and 2‐methoxydiphenylether, yielding the corresponding products. These results unequivocally demonstrated that the presence of electron‑donating methoxy groups did not hinder the catalytic performance of NiAlO_x_‐300, and highlighted the excellent substrate adaptability and robustness of the NiAlO_x_‐300 catalyst. The capability of catalyzing hydrogenolysis of various lignin models at relatively lower reaction temperatures confirmed the superior activity of NiAlO_x_‐300. Additionally, considering that many aryl C‐O linkages were contained in real lignin, NiAlO_x_‐300 was employed to catalyze the hydrogenolysis of real lignin at 200°C with a reaction time of 4 h and a H_2_ pressure of 2 MPa. Although the real lignin could be indeed converted, the overall monomer yield was still low (ca. 5 wt%). Thereby, the NiAlO_x_‐300 should be further optimized for efficiently converting real lignin, which were undergoing in our lab.

**TABLE 2:** Catalytic hydrogenolysis of various lignin models over NiAlO_x_‐300.^[a]^

Entry	Substrate	Time (h)	Temperature (°C)	Conversion (%)^[b]^	Yield (%)^[b]^
1	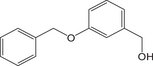	5	50	100	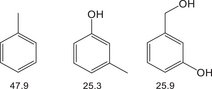
2	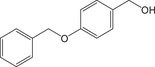	5	50	100	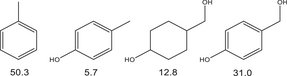
3	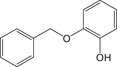	5	50	100	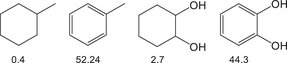
4	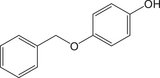	5	50	100	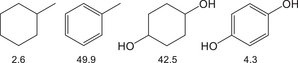
5	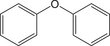	5	100	100	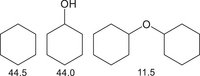
6	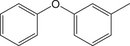	7	100	100	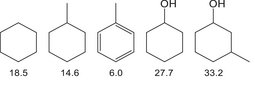
7	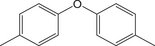	7	100	100	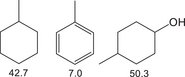
8	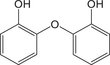	7	100	100	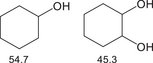
9	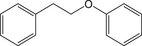	5	80	100	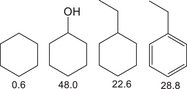
10	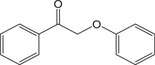	5	100	100	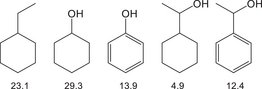
11	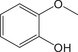	7	120	100	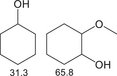
12	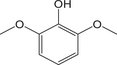	5	150	100	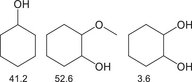
13	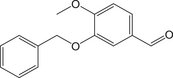	5	50	100	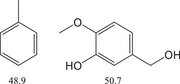
14	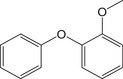	5	150	100	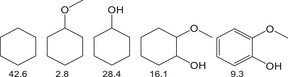

^[a]^ Reaction conditions: substrate, 1.0 mmol; ethanol, 5 g; NiAlO_x_‐300, 45 mg.

^[b]^ Conversion and yield were determined by GC using *n*‐dodecane as the internal standard.

### 2.5 Reasons for Excellent Catalytic Activity of NiAlO_x_‐300

As discussed above, NiAlO_x_‐300 displayed excellent catalytic performance in the hydrogenolysis of lignin models at mild reaction conditions. It is crucial to discuss the reason on the superior catalytic activity of NiAlO_x_‐300. Based on the XRD and XPS results (Figure 2a,c), metallic Ni was generated in all four NiAlO_x_‐T samples. Generally, metallic Ni can activate H_2_, and thus has been widely applied in hydrogenation reactions [56, 63]. Thus, metallic Ni was probably the catalytically active site for the hydrogenolysis of various lignin models. However, as described in XPS spectra (Figure 2c), the content of metallic Ni increased with the improved reduction temperature to prepare the NiAlO_x_‐T materials, which was opposite with their catalytic activity. Meanwhile, as examined by CO‐pulse titration technique, the four NiAlO_x_‐T materials displayed similar Ni dispersion (Table S7), which could exclude the influence of Ni dispersion on catalytic activity. According to these discussions, it could be deduced that there should be some other aspects to enable hydrogenolysis of lignin models to occur at milder reaction temperatures (30°C–120°C) over the NiAlO_x_‐300 catalyst.

As is well‐known, solid surface frustrated Lewis pairs (ssFLPs) can act as active sites to activate small molecules like H_2_ [64]. Previous studies have revealed that ssFLPs could be created by a coordinately unsaturated surface metal site (as the Lewis acidic site) adjacent to an oxygen vacancy and a Lewis basic surface hydroxide site (OH_surface_) [65]. First, as examined by electron paramagnetic resonance (EPR), all the synthesized NiAlO_x_‐T catalysts exhibited a distinct defect signal of oxygen vacancies at g = 2.003 (Figure S9), indicating there were abundant oxygen vacancies on the constructed NiAlO_x_‐T catalysts. Second, as described in XPS spectra of Al 2p (Figure S4), unsaturated Al^3+^ species (Al^3+^_unsatur._) were detected in the NiAlO_x_‐T catalysts. Third, considering that the NiAlO_x_‐T catalysts were constructed from NiAl‐LDHs precursor at a reduction temperature of below 600°C, abundant surface hydroxides were reserved. In addition, as previously reported, ssFLPs could be successfully constructed by thermal treatment of AlOOH [41]. Based on these discussions above, it could be deduced that ssFLPs (Al^3+^_unsatur._‐OH_surface_) were created by the reduction of NiAl‐LDHs precursor. Probably, these formed ssFLPs played a crucial role in affecting the catalytic activity.

**FIGURE 4:**
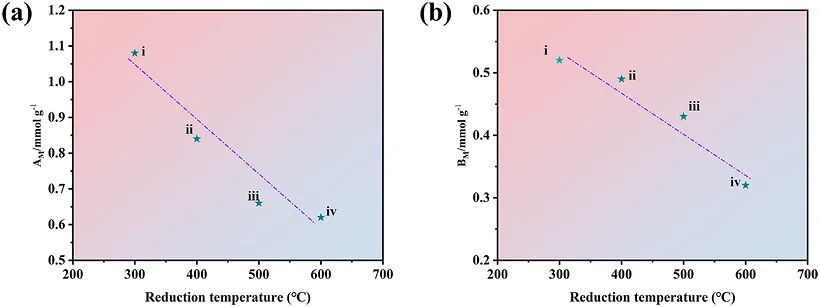
(a) Correlation between A_M_ and reduction temperature, and (b) correlation between B_M_ and reduction temperature ratio. (i) NiAlO_x_‐300, (ii) NiAlO_x_‐400, (iii) NiAlO_x_‐500, and (iv) NiAlO_x_‐600.

Considering the crucial role of the acid–base properties in hydrogenolysis of lignin models, the concentration of base/acid sites on the prepared NiAlO_x_‐T catalysts was determined by widely used CO_2_‐TPD and NH_3_‐TPD techniques. For all four NiAlO_x_‐T samples, a broad desorption peak was observed between 100°C and 700°C in the spectra of both CO_2_‐TPD and NH_3_‐TPD (Figure S10). To obtain the strength and concentration of basic and acidic sites, the CO_2_‐TPD and NH_3_‐TPD profiles were deconvoluted into three peaks according to the desorption temperature via a Gaussian peak fitting method (Table S3), and the desorption temperature and area of fitted peaks for CO_2_‐TPD and NH_3_‐TPD corresponded to the strength and quantity of base/acid sites, respectively. In CO_2_‐TPD profiles, the deconvoluted three desorption peaks appeared in the regions of 220°C–250°C, 310°C–340°C, and 450°C–480°C, which were identified as the weak (B_W_), medium‐strong (B_M_), and strong base (B_S_) sites, respectively. According to previously reported results, the strong basic sites in NiAlO_x_‐T catalysts originated from low‐coordinated oxygen anions. Meanwhile, NH_3_‐TPD analysis revealed three distinct desorption peaks at 220°C–260°C (weak acid sites, A_W_), 420°C–460°C (medium‐strong acid sites, A_M_), and 620°C–640°C (strong acid sites, A_S_) [66, 67, 68, 69]. This provides stronger evidence for the simultaneous presence of both acidic and basic sites on the catalyst, which further supports the existence of solid surface frustrated Lewis pairs (ssFLPs) on the catalyst surface. Notably, the concentration of medium‐strong base or acid sites decreased almost linearly with the increment of reduction temperature in the NiAlO_x_‐T catalysts (Figure 4), indicating the association of medium‐strong acid/base sites with the NiO‐like phase. Besides, the increment in strong acid sites originated from the formation of bridged hetero metal‐oxygen bonds (e.g., Ni─O─Al bonds), which could induce lattice contraction in NiO‐like phases and create distorted octahedral Ni─O coordination. Interestingly, with the increase in reduction temperature, strong basic sites emerged significantly on both the 500°C and 600°C reduced samples. This phenomenon can be attributed to the generation of a greater number of oxygen vacancies on the catalyst surface induced by the higher reduction temperature, which significantly increases the electron cloud density of the lattice oxygen ions (O^2−^) [70]. This structural modification enhanced electron density around O^2−^ (Lewis base sites) while reducing electron density at Ni^2+^ centers (Lewis acid sites), synergistically modulating the catalyst's acid‐base properties [54, 68].

As described in Table 1 and Table S3, the catalytic activity of the NiAlO_x_‐T catalysts positively correlated with their medium‐strength acid and base sites. First, pyridine‐adsorbed infrared spectroscopy (Py‐IR) was performed to elucidate the nature of acid sites on the surface of the NiAlO_x_‐T catalysts. In the Py‐IR spectra (Figure S11), there were characteristic peaks appearing at 1440 cm^−1^ and 1610 cm^−1^, indicating the presence of abundant Lewis acid sites [35, 71], which could be attributed to Lewis acid sites [72]. It could be speculated that the contents of Lewis acid sites in the NiAlO_x_‐T catalysts followed the order: NiAlO_x_‐300 > NiAlO_x_‐400 > NiAlO_x_‐500 > NiAlO_x_‐600 (Table 1). Second, the content of the surface hydroxides decreased with the thermal treatment temperatures [41], and the surface hydroxides often acted as Lewis base sites [73]. Obviously, the change of the content of Lewis base sites in the NiAlO_x_‐T catalysts followed the same order with that of the contents of Lewis acid sites. Thereby, the content of ssFLPs (Al^3+^_unsatur._‐OH_surface_) in the four NiAlO_x_‐T catalysts decreased with the increase of reduction temperature, and their catalytic activity showed a positive correlation with the content of the ssFLPs (Al^3+^_unsatur._‐OH_surface_). Nevertheless, based on the results in Table 1, both Ni/NiO (Table 1, entry 6) and AlO_x_(OH)_y_‐300 (Table 1, entry 7) were inactive for the hydrogenolysis of lignin models, indicating that both Ni and Al active species were indispensable to drive the hydrogenolysis of lignin models. However, the catalytic activity of the prepared NiAlO_x_‐T catalysts decreased with the increased reduction temperature to prepare them, implying that a small amount of metallic Ni° could cooperate with the ssFLPs. Probably, the metallic Ni^0^ acted as the active sites for activating H_2_ to form Ni‐H species [33], which were coupled to the ssFLPs (Al^3+^_unsatur._‐OH_surface_) sites to participate in the hydrogenolysis process [73]. The significantly low activity of NiAlO_x_‐600 originated from the destruction of the ssFLPs at 600°C. Thereby, we deduced that the properties of the formed ssFLPs (i.e., Al^3+^_unsatur._‐OH_surface_) played a more crucial role in promoting hydrogenolysis of lignin models at mild reaction conditions. To confirm whether the ssFLPs sites participated in the hydrogenolysis process or not, control experiments with benzoic acid (to poison the Lewis basic sites) and pyridine (to poison the Lewis acidic sites) as the additives were performed. It was observed that the hydrogenolysis was completely quenched (Table 1, entries 10 and 11), verifying that the ssFLPs sites indeed attended the hydrogenolysis process. Additionally, oxygen vacancy could abstract the O atom in C─O bonds, playing a pivotal role in the hydrogenolysis of C─O bonds [74]. As displayed in Figure S9, the relative intensity of oxygen vacancies progressively increased with the increase of reduction temperature to prepare the catalyst, indicating that higher reduction temperatures facilitated the formation of more oxygen vacancies. However, the catalytic performance did not improve monotonically with increasing oxygen vacancy concentration. Especially, the NiAlO_x_‐600 with the highest oxygen vacancy density exhibited the poorest activity because excessive oxygen vacancies would result in overly strong adsorption of reactants, which would cover the catalytically active sites, resulting in deterioration of catalytic efficiency. Furthermore, as shown in Figure S10, it is intriguing to observe that NiAlO_x_‐500 and NiAlO_x_‐600 possess nearly identical basicity. However, the sample reduced at 600°C exhibits significantly stronger acidic sites, yet its catalytic activity decreased markedly. This indicates that, in contrast to strong basic sites, the presence of strong acidic sites may be a critical factor inhibiting the reaction in this work. Based on the discussions above, it could be concluded that the excellent activity of NiAlO_x_‐300 originated from the good synergistic effect between ssFLPs (Al^3+^_unsatur._‐OH_surface_) sites and the metallic Ni^0^. The ssFLPs sites were probably the central catalytically active sites, while the metallic Ni^0^ acted like an initiator.

### 2.6 Mechanistic Investigation

**SCHEME 1:**
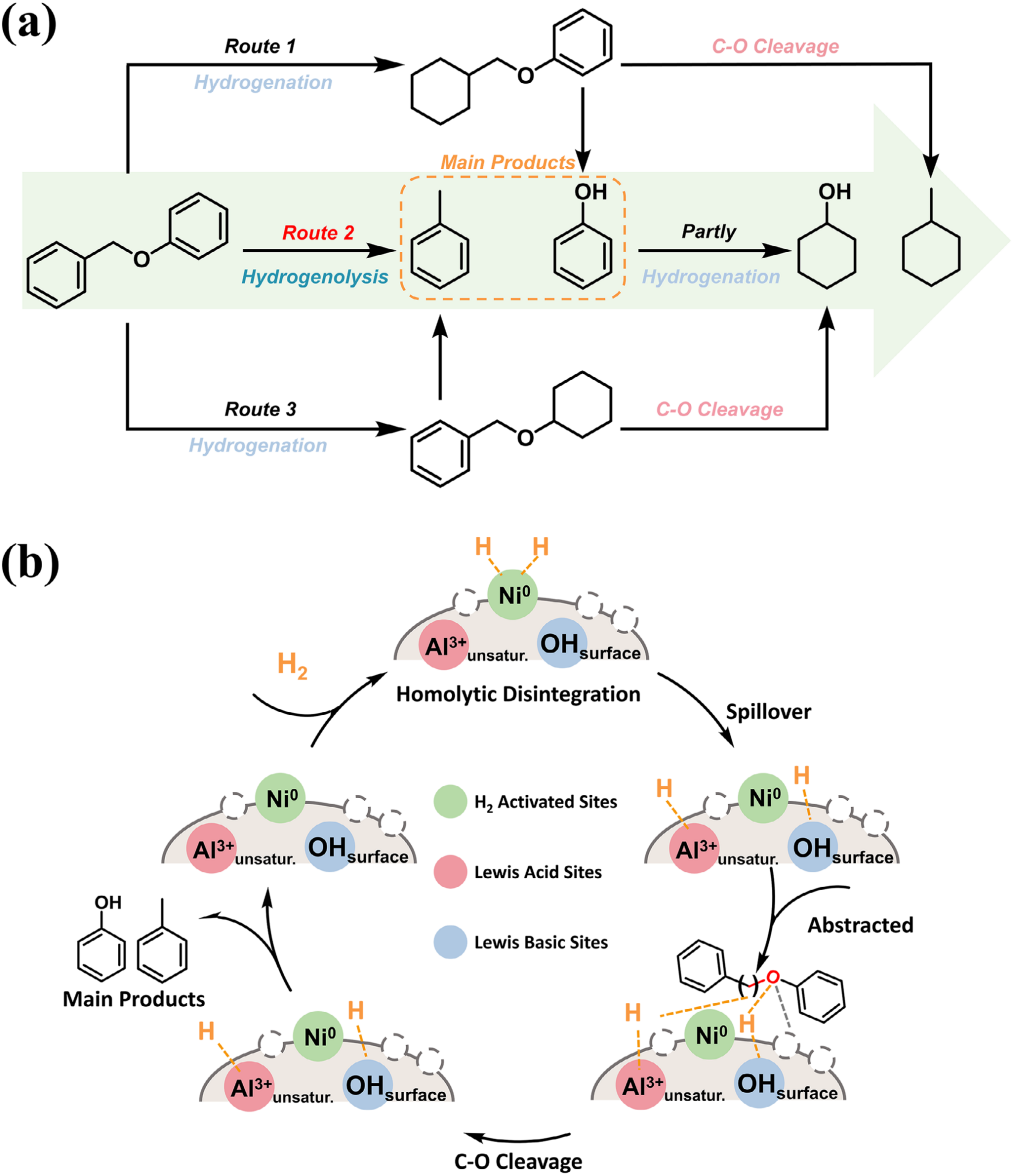
(a) Proposed reaction pathway for hydrogenolysis of benzyl phenyl ether, and (b) Proposed mechanism of hydrogenolytic cleavage of benzyl phenyl ether over the fabricated NiAlO_x_‐T catalyst.

Generally, hydrogenolysis of benzyl phenyl ether proceeded through two different reaction pathways [34], including (i) direct hydrogenolysis of the C─O bond, and (ii) hydrogenation of one benzene ring and subsequent hydrogenolysis of the C─O bond. Based on the results above, toluene and phenol were the two main products at the reaction temperature of 30°C, while the products from hydrogenation of one benzene ring (i.e., [(cyclohexyloxy)methyl] benzene or (cyclohexylmethoxy)benzene, in Routes 1 and 3 of Scheme 1a) were nearly undetected. These results suggested that the predominant reaction pathway was direct hydrogenolysis to generate toluene and phenol (Route 2 in Scheme 1a). Additionally, it was also observed that methylcyclohexane and cyclohexanol would be generated at a high reaction temperature (e.g., 100°C).

Based on the experimental results and some reported knowledge [33, 74, 75], a plausible reaction mechanism was proposed for the NiAlO_x_‐300‐catalyzed hydrogenolysis of benzyl phenyl ether (Scheme 1b). Molecular hydrogen was adsorbed and activated by the metallic Ni^0^ sites at 30°C, resulting in the generation of active Ni‐H species. Then, the H atom in the generated Ni‐H species migrated to the ssFLPs (Al^3+^_unsatur._‐OH_surface_) sites via hydrogen spillover, and became coupled to the Al^3+^_unsatur._ site and the OH_surface_ site [73], respectively. Simultaneously, the O atom in C─O bond was abstracted by the abundant oxygen vacancies on NiAlO_x_‐300, and thus the ether bond was activated. Finally, toluene and phenol were produced through the catalytic C─O cleavage by active H species on ssFLPs sites. Under mild reaction conditions (e.g., 30°C), toluene and phenol were difficult to be further hydrogenate to form methylcyclohexane and cyclohexanol.

## 3 Conclusion

In conclusion, the NiAlO_x_‐300 catalytic material was constructed by the treatment of NiAl‐LDH precursor with H_2_ at 300°C, producing abundant surface FLP sites (Al^3+^_unsatur._‐OH_surface_) and suitable metallic Ni^0^. It was shown that the NiAlO_x_‐300 could efficiently catalyze the hydrogenolysis of various lignin model compounds containing α‐O‐4, β‐O‐4, and 4‐O‐5 linkages under mild conditions (below 100°C, 1 MPa H_2_). Especially, the α‐O‐4 models could be converted at a temperature of ≤ 50°C (only 20°C for benzyl phenyl ether). Systematic study revealed that the excellent performance of NiAlO_x_‐300 originated from the synergistic effect of the generated ssFLP sites (Al^3+^_unsatur._‐OH_surface_) and metallic Ni^0^. The metallic Ni^0^ sites activated molecular hydrogen to form Ni‐H species, while the ssFLP sites (Al^3+^_unsatur._‐OH_surface_) coupled with the H atom in the active Ni‐H species to form active hydrogen species, which reacted with the lignin modes to generate the corresponding products. Moreover, mechanistic investigation confirmed that direct cleavage of the C─O bond was the primary reaction pathway for hydrogenolysis of benzyl phenyl ether at 30°C. The discovery of the synergy between metallic non‐noble‐metal sites and solid surface frustrated Lewis pairs provides new opportunities to design efficient catalysts for the valorization of lignin and lignin derivatives.

## Conflicts of Interest

The authors declare no conflict of interest.

## Supporting information




**Supporting File**: advs75820‐sup‐0001‐SuppMat.doc.

## Data Availability

The authors have nothing to report.
