# Supplementary material for: Frustrated Lewis Pairs and Metallic Ni Synergistically Enabled Low‐Temperature Hydrogenolysis of Lignin Models
Source: Adv Sci (Weinh). 2026 May 25:e20585. Online ahead of print. doi: 10.1002/advs.202520585 (PMC13335851; doi:10.1002/advs.202520585)
Supplement: Supplementary file 1 — Supporting File: advs75820‐sup‐0001‐SuppMat.doc. [file ADVS-9999-e20585-s001.doc]

*Supporting Information for*

**Frustrated Lewis Pairs and Metallic Ni Synergistically Enabled Low-temperature Hydrogenolysis of Lignin Models**

Jinpeng Liang,^[a]^ Ziyi Ma,^[a]^ Zhaoxi Cai,^[a]^ Kongqian Liang,^[a]^ Chenglei Xiao,^[a]^ Sitong Chen,^[a]^ Na Wang,^[a]^ Zhimin Xue,*^[b]^ Chao Xie,*^[c]^ and Jinliang Song^*[a]^

[a] J. Liang, Z. Ma, Z. Cai, K. Liang, C. Xiao, S. Chen, N. Wang, Prof. J. Song
School of Chemical Engineering and Light Industry
Guangdong University of Technology
Guangzhou 510006, China
E-mail: songjl_2021@gdut.edu.cn

[b] Prof. Z. Xue
State Key Laboratory of Efficient Production of Forest Resources, College of Materials Science and Technology
Beijing Forestry University
Beijing 100083, China

E-mail: zmxue@bjfu.edu.cn

[c] Dr. C. Xie
National & Local Joint Engineering Research Center on Biomass Resource Utilization, College of Environmental Science and Engineering
Nankai University
Tianjin 300350, China

E-mail: xiechao@nankai.edu.cn

**Experimental Section**

***Chemicals***. Nickel nitrate hexahydrate (Ni(NO_3_)_2_·6H_2_O, 99%), copper nitrate hexahydrate (Cu(NO_3_)_2_·3H_2_O, 99%), and NaOH (99%) were bought from Guangzhou Chemical Ltd. Aluminum nitrate nonahydrate (Al(NO_3_)_3_·9H_2_O, 99%), cobalt nitrate hexahydrate (Co(NO_3_)_2_·6H_2_O, 99%), and Na_2_CO_3_ were purchased from Aladdin company. Ni(OH)_2_ (61 wt% Ni), 2’-phenoxyacetophenone (97%), phenoxyethylbenzene (97%), 4-(benzyloxy)phenol (97%), 2-(benzyloxy)phenol (97%), methanol (99.7%), propanol (99.5%), 1-butanol (99.5%), *n*-haxane (99%), and ethanol (99.9%) were supplied by Macklin. Diphenyl ether (99%), and isopropanol (99.9%) were purchased from J&K Scientific Ltd. The above chemicals were used without any further purification. Besides, we should point out that the water used in this work was deionized water.

***Materials synthesis****.* The pristine NiAl-LDH (Ni/Al feeding ratio of 3/1) was initially synthesized as the catalyst precursor by a hydrothermal process. Typically, an aqueous solution containing the corresponding amounts of Ni(NO_3_)_2_⋅6H_2_O and Al(NO_3_)_3_⋅9H_2_O was prepared. Meanwhile, a basic aqueous solution containing NaOH and Na_2_CO_3_ with the molar ratios of [CO_3_^2−^]/[Al^3+^]=2.0 and [OH^-^]/[Co^2+^+Al^3+^]=1.6 was obtained by dissolving the corresponding amount of NaOH and Na_2_CO_3_ in deionized water. Specifically, 3.490 g of Ni(NO_3_)_2_⋅6H_2_O and 1.500 g of Al(NO_3_)_3_⋅9H_2_O were dissolved in 40 mL of deionized water under stirring to form solution A. Meanwhile, 0.256 g of NaOH and 0.848 g of Na_2_CO_3_ were dissolved in 40 mL of deionized water to form solution B. Then, solution A and solution B were transferred into two constant pressure dropping funnels, respectively. Under continuous magnetic stirring at room temperature, these two solutions were simultaneously added dropwise at a constant rate into a 120 mL pressure-resistant bottle, and the pH value of the solution was maintained at approximately 9.0. The obtained turbid liquid was then stirred at 80 °C for 24 h. Thereafter, the precipitation was filtered, thoroughly washed until the pH value of the washing water reached 7.0 and then dried at 80 °C for at least 24 h. Finally, the samples were heated at constant heating rate of 5 °C min^-1^ up to 300 °C and maintained for 2 h at pure H_2_ flow, and the obtained material was denoted as NiAlO_x_-300. The pristine NiAl-LDH nanosheets are then reduced in the H_2_ flow for 2h at different temperatures (400 °C: NiAlO_x_-400; 500 °C: NiAlO_x_-500; 600 °C: NiAlO_x_-600). In comparison, Ni/NiO and AlO_x_(OH)_y_-300 were prepared by reduction of Ni(OH)_2_ and AlOOH (prepared by a reported in-situ growth technique^[1]^) at 300 °C.

***Synthesis of MAl-LDH and MAlO_x_-300.*** MAl-LDH and MAlO_x_-300 catalysts were prepared using the same method described above except that nickel nitrate was replaced by the corresponding metal nitrates.

***Hydrogenolysis of lignin models.*** Typically, the examined substrate (1 mmol), the desired catalyst (45 mg), and ethanol (5 g) were added to a reactor. After the reactor was sealed, the inside of the reactor needed to be repeatedly flushed with high-purity H_2_ at least 5 times. Afterward, the autoclave was charged with 1 MPa of H_2_. The reaction was conducted at the desired temperature with a stirring speed of 600 rpm. After the reaction, the reactants and products were analyzed by a Shimadzu GC-2010 instrument equipped with a flame ionization detector using *n*-dodecane as the internal standard.

***Reusability of the NiAlO_x_-300.*** To evaluate the reusability of NiAlO_x_-300, the used catalyst was separated from the reaction solution by centrifugation, and washed carefully with ethanol (3 × 5 mL). Subsequently, the recovered NiAlO_x_-300 was used for the next catalytic cycle by adding new reactants and the solvent. In addition, to exclude the influence of the “dirty” stir bar on the catalytic performance, all the used stir bars were treated by aqua regia for 48 h to thoroughly remove the residues adsorbed on the stir bars.

***Examination and calculation methods for reactant conversion and product yield.*** The products were analyzed by gas chromatography (GC) equipped with a flame ionization detector and a WondaOap-5 capillary column (0.53 mm diameter, 30 m length, GL Sciences). In addition, the products were identified by gas chromatography-mass spectrometry (GC-MS) using an Agilent 8890 gas chromatograph (fitted with an HP-5MS UI capillary column, 30 m × 0.25 mm I.D. × 0.25 μm film thickness) and an Agilent 5977C mass spectrometer. First, the GC-FID analysis conditions were as follows: column type (HP-5, 30 m × 0.32 mm × 0.25 μm), temperature program (initial 40 ^o^C, hold 2 min; ramp to 300 ^o^C at 10 ^o^C/min, hold 5 min), injector and detector temperatures (300 ^o^C), split ratio (20:1), and carrier gas (N_2_, 225 mL/min). Second, for low-boiling alkanes (e.g., methylcyclohexane, cyclohexane, and toluene), we used a solvent delay of 2.5 min to avoid solvent peak interference. The retention times of all products were confirmed by injecting authentic standards. Third, the quantification method: internal standard (n-dodecane) was added after the reaction, and calibration curves (R² > 0.999) were established for each product over a concentration range of 0.1-10 mmol/L. Finally, for alkane products with very low boiling points (e.g., methane, and ethane) that are not retained under our GC conditions, we used GC-MS to determine them. However, under our mild reaction conditions (30 ^o^C), no such light alkanes were detected. The conversions and yields were calculated based on the following formulas:

Conversion of the reactant (*X*):
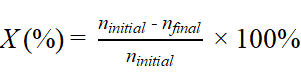


Herein, *n_initial_* = Initial mole of the reactant, and *n_final_* = Unreacted mole of the reactant).

Yield of the product (*Y_p_*):
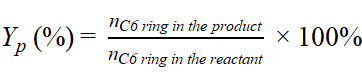


Herein, *n_C6 ring in the product_* = Mole of C6 ring in the product, and *n_C6 ring in the reactant_* = Mole of C6 ring in the reactant.

***Characterization techniques****.* The surface area of each sample was determined from the N_2_ adsorption isotherm obtained at 77 K using the Brunauer-Emmett-Teller (BET) method with a Micromeritics ASAP 2460. Transmission electron microscopy (TEM, JEM-2100F, JEOL) was used to determine the particle size and dispersion of the catalysts. Powder X-ray diffraction (XRD) patterns were collected on a Rigaku MiniFlex600 X-ray diffractometer using Cu Kα radiation (λ =1.5418 Å). All catalysts were measured at a scanning speed of 10°/min from 10 to 80°. XPS analysis was performed on an Thermo K-Alpha (America Thermo) using Al Kα X-ray radiation source (1486.6 eV photons) with a power of 150 W. Thermogravimetric analysis (TGA) was done using a Netzsch Germany STA449F3 instrument in N_2_ atmosphere at a heating rate of 10 °C min^-1^ from room temperature to 800 °C. The pyridine-FTIR analysis was performed on Bruker Tensor-27, and the spectrum was recorded from 373 to 573 K. The EPR data were gained from Germany Bruker A300-10/12). H_2_ temperature programmed reduction (H_2_-TPR) were conducted using a Altamira Instruments AMI-300 with a thermal conductivity detector (TCD). About 100 mg of sample was loaded in a quartz reactor, and then TPR was carried out with a heating ramp rate of 10°C min^-1^ in a gaseous mixture of H_2_ and He (1/9, v/v) to a sample temperature of 900°C, with a total flow rate of 50 mL·min^−1^. Before TPR measurement, 50 mg of catalyst was loaded to a quartz fixed-bed U-shaped micro-reactor (i. d.=4mm) and pretreated in an H_2_ flow of 30 mL·min^−1^ at 300 to 600 °C for 2 h. After being cooled in Ar atmosphere to 50 °C and then kept with an Ar flow of 30 mL·min^-1^ for 1h, the pretreated sample was finally exposed to a flow (30 mL·min^−1^) of 10% H_2_/Ar mixture and heated from 50 to 900 °C at a ramp of 10 °C·min^-1^. CO_2_ temperature programmed desorption (CO_2_-TPD) were carried out on a chemical adsorption analyzer (tp-5080, China Xianquan). The acidity of the tested catalysts was detected by NH_3_ temperature-programmed desorption (NH_3_-TPD) in quartz tubular reactor in an autocatalytic adsorption system (Zetasizer Nano ZS90, England Malvern). About 100 mg of sample for either CO_2_-TPD or NH_3_-TPD test was first reduced at 300 °C for 1 h in 10 vol % H_2_/He, followed by flushing (50 mL·min^-1^) with high purity He for 1 h at 50 °C. Afterward, as the temperature decreased to 100 °C, either CO_2_ or NH_3_ was injected until saturation. CO_2_-TPD or NH_3_-TPD was carried out with a heating ramp rate of 10 °C min^-1^ to a sample temperature of 500 °C.


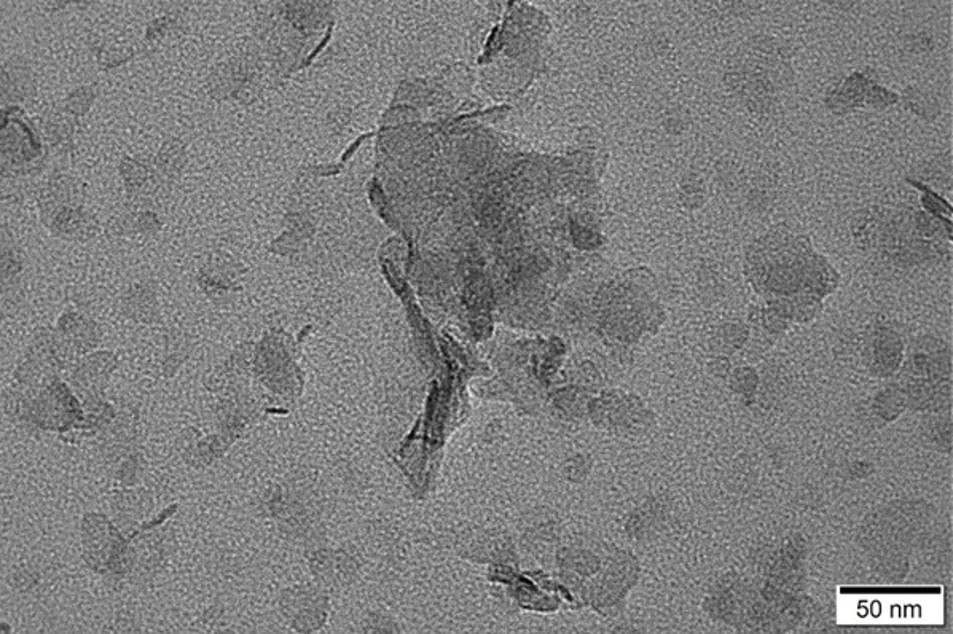


**Figure S1.** TEM image of NiAl-LDH precursor.


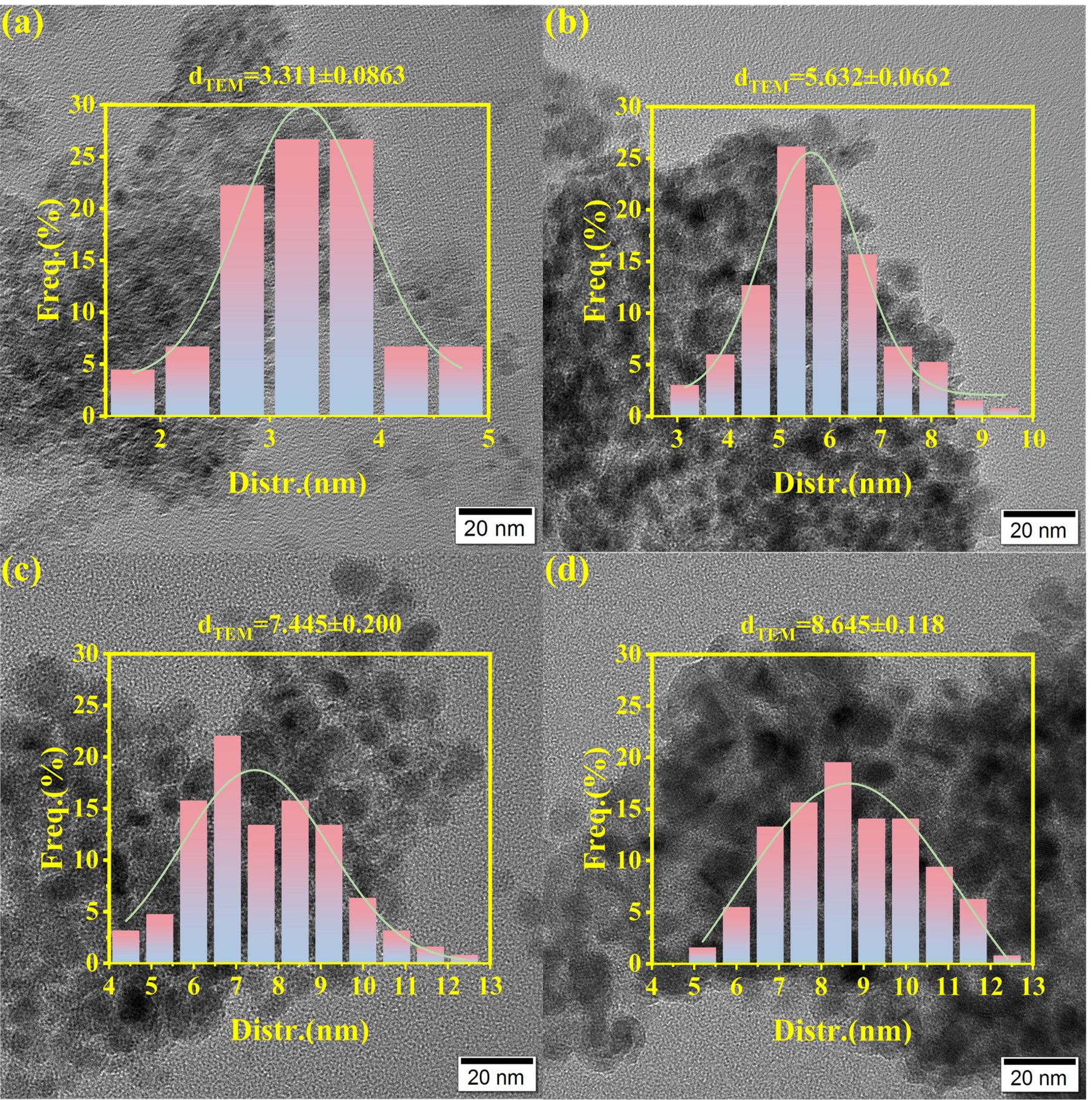


**Figure S2.** TEM images of (a) NiAlO_x_-300, (b) NiAlO_x_-400, (c) NiAlO_x_-500, and (d) NiAlO_x_-600.


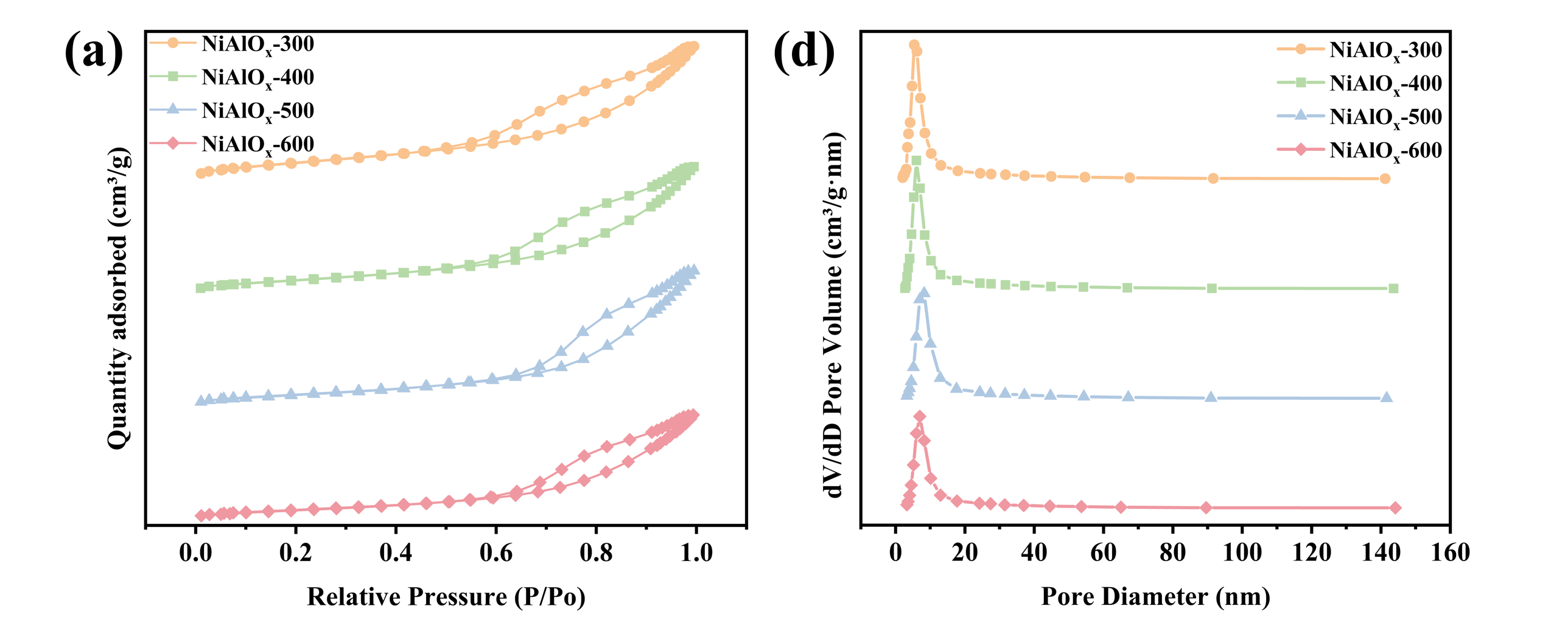


**Figure S3.** (a) N_2_ adsorption/desorption isotherms of NiAlO_x_-T catalysts, and (b) corresponding Barrett-Joyner-Halenda (BJH) pore-size distribution curve.





**Figure S4.** XPS spectra of Al 2p.





**Figure S5.** H_2_-TPR profiles of NiAlO_x_-T.





**Figure S6.** XRD patterns of fresh NiAlO_x_-300 and the spent NiAlO_x_-300 catalysts.


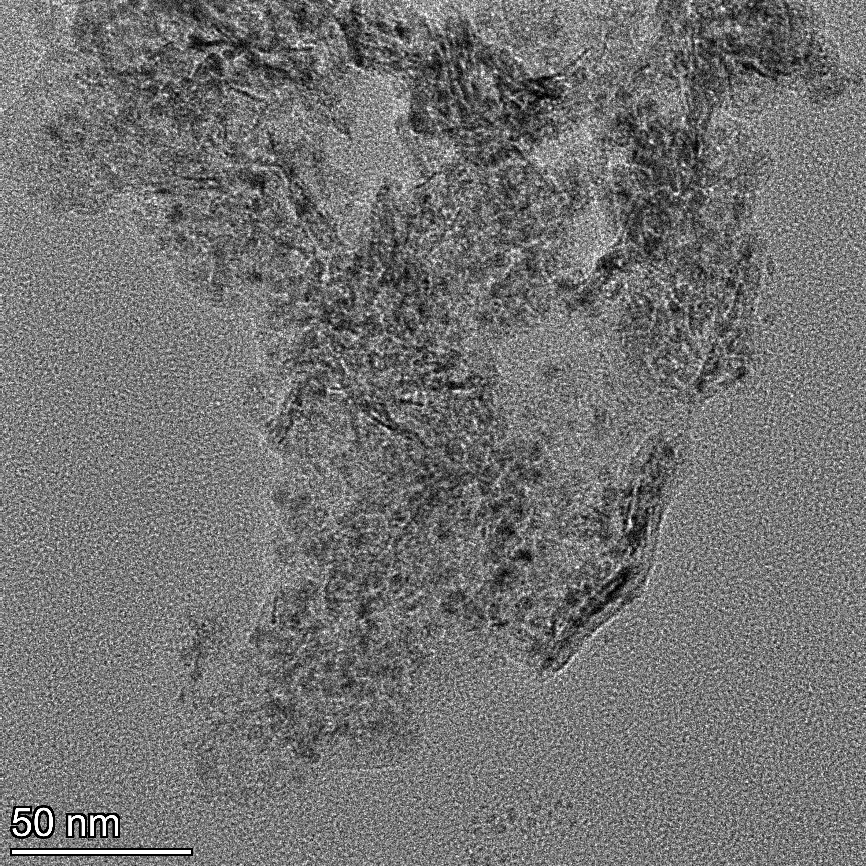


**Figure S7.** TEM images of the spent NiAlO_x_-300 catalyst.





**Figure S8.** Time-conversion plots for hydrogenolysis of benzyl phenyl ether over NiAlO_x_-300. Reaction conditions: Benzyl phenyl ether, 1.0 mmol; ethanol, 5 g; reaction temperature, 30 °C; reaction time, 5 h; 1 MPa H_2_.


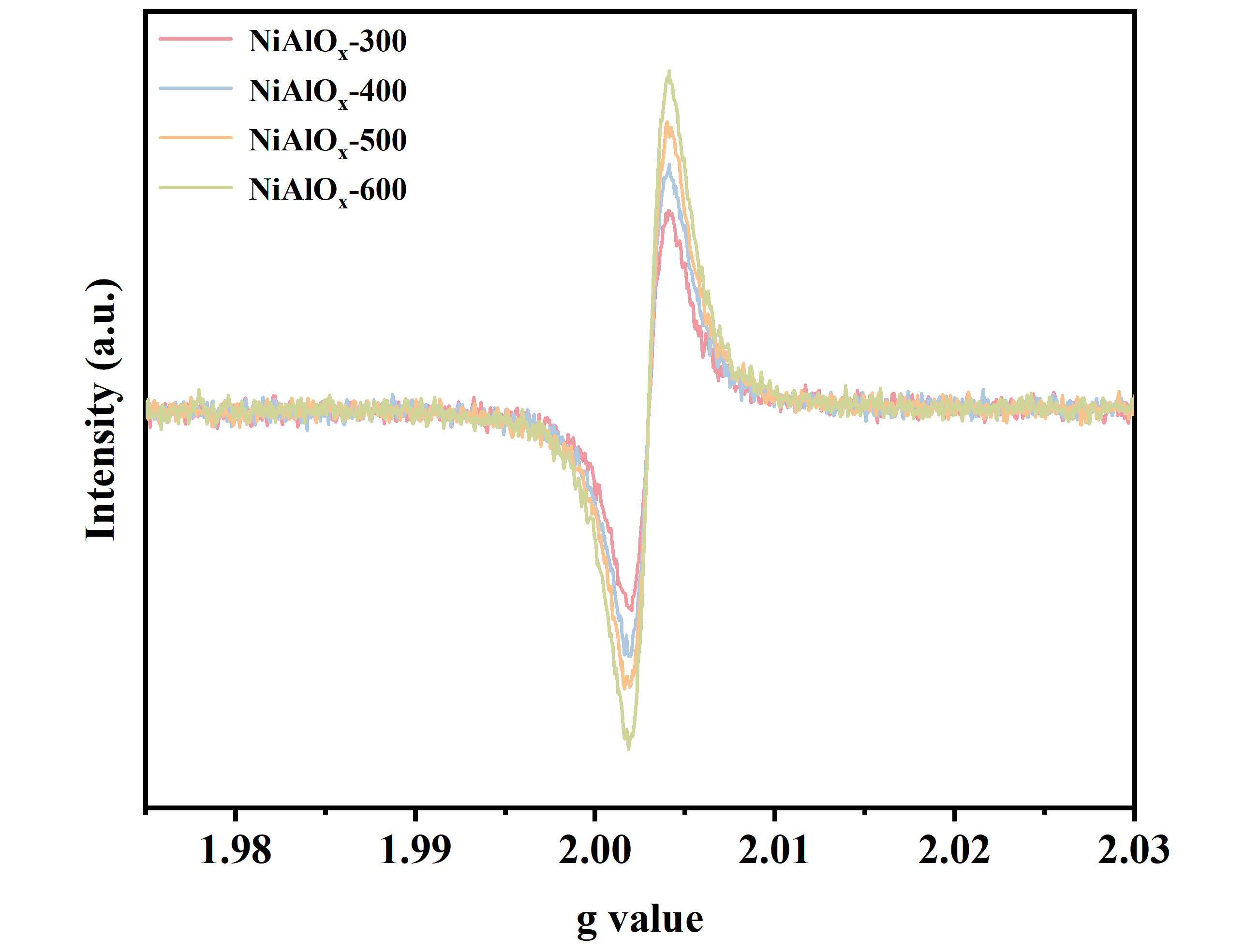


**Figure S9.** EPR spectra of the synthesized NiAlO_x_-T catalysts.

**
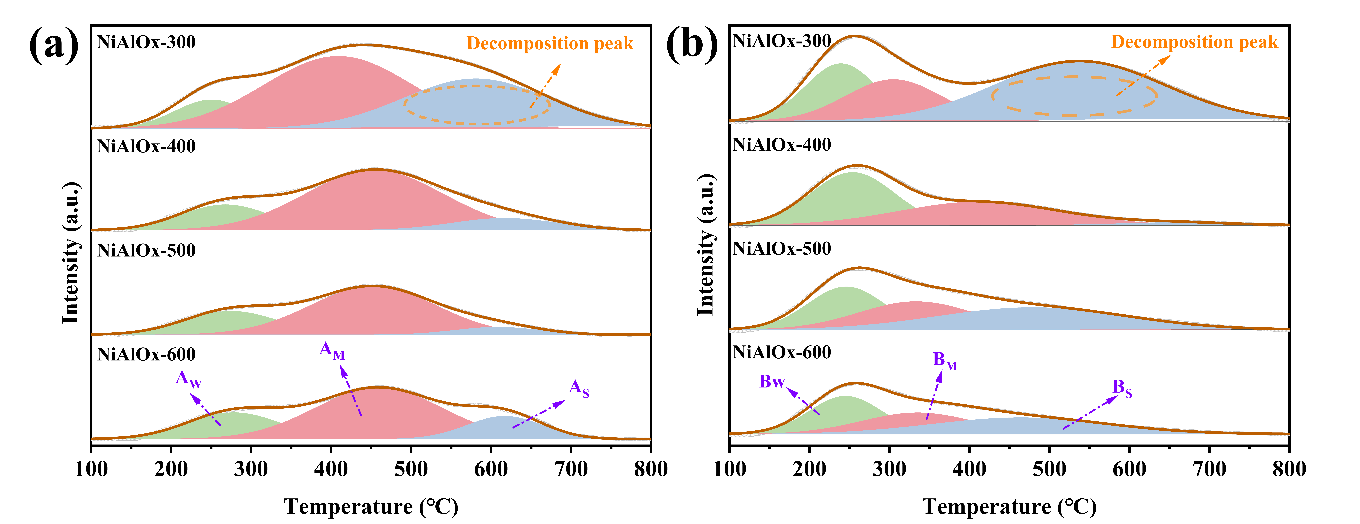
**

**Figure S10.** (a) NH_3_-TPD profiles, which were deconvoluted into three peaks assigned to weak acid (A_W_), medium-strong acid (A_M_) and strong acid (A_S_) site. (b) CO_2_-TPD profiles, which were deconvoluted into three peaks corresponding to weak base (B_W_), medium-strong base (B_M_) and strong base (B_S_) site.

**
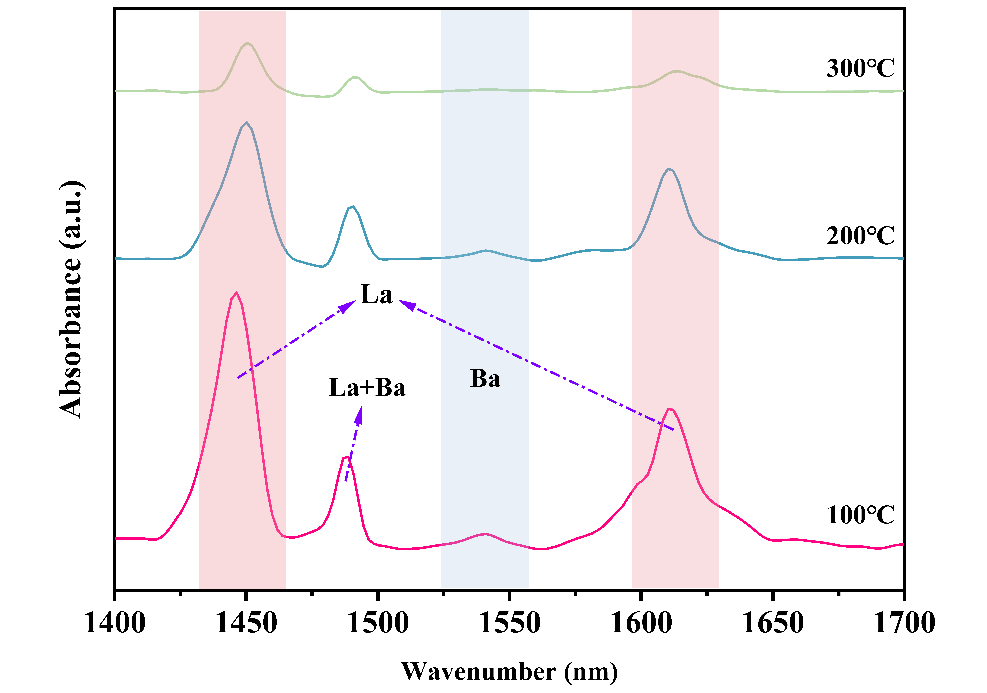
**

**Figure S11.** Py-FTIR spectra of NiAlO_x_-300 at 100 to 300 °C.


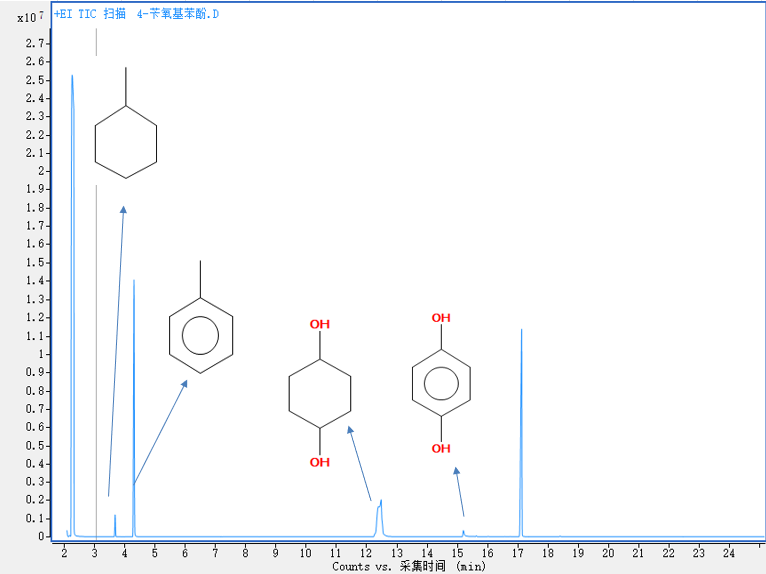


**Figure S12.** Raw GC-MS chromatogram of products from the hydrogenolysis of 4‑phenoxyphenol (C_α_-O-Ar linkage) at 50 ^o^C.


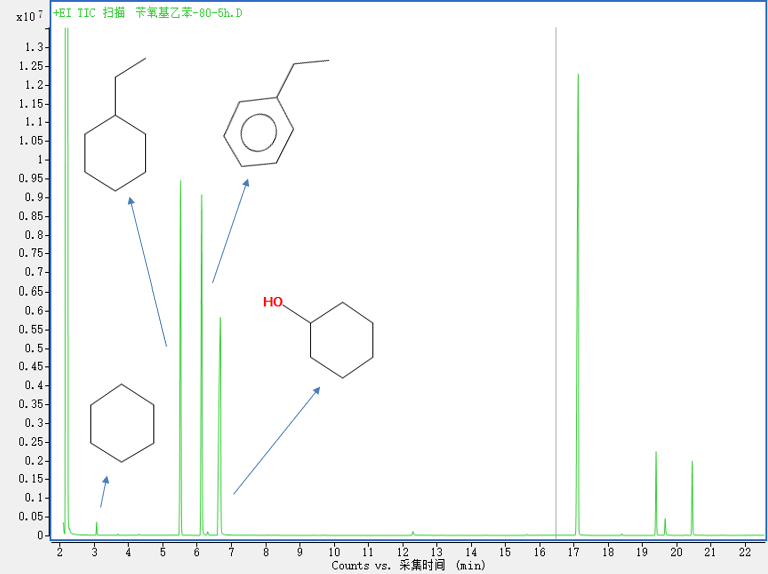


**Figure S13.** Raw GC-MS chromatogram of products from the hydrogenolysis of 2-phenoxy-1-phenylethanol (C_β_-O-Ar linkage) at 80 ^o^C.


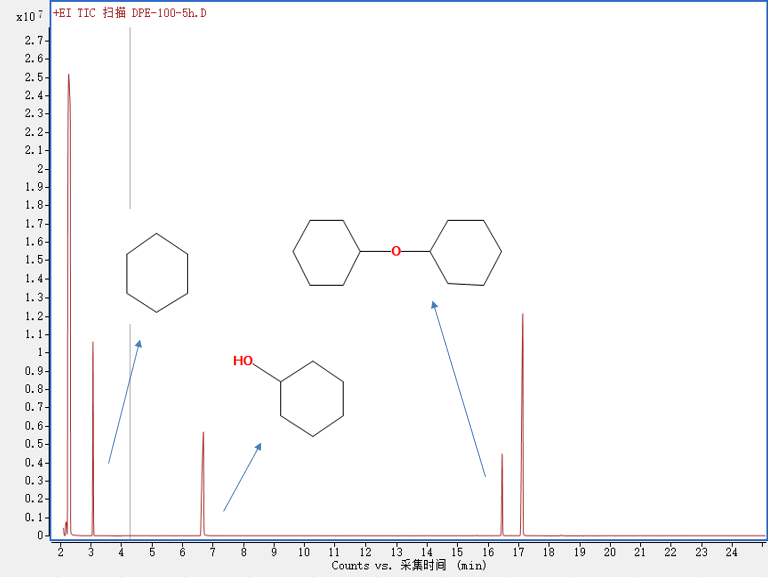


**Figure S14.** Raw GC-MS chromatogram of products from the hydrogenolysis of diphenyl ether (Ar-O-Ar linkage) at 100 ^o^C.

**Table S1.** Physicochemical properties of the fabricated NiAlO_x_-T materials.

| **Sample** | **Ni content (wt%)^[a]^** | **Al content (wt%)^[a]^** | **Ni particle size (nm)^[b]^** | **Surface area (m^2^/g)^[c]^** | **Pore volume (cm^3^/g)^[d]^** | **Pore size (nm)^[e]^** |
| --- | --- | --- | --- | --- | --- | --- |
| NiAlO_x_-300 | 56.19 | 9.36 | 3.31±0.086 | 200.9 | 0.46 | 9.31 |
| NiAlO_x_-400 | 60.28 | 10.01 | 5.62±0.066 | 158.5 | 0.43 | 10.60 |
| NiAlO_x_-500 | 61.46 | 10.15 | 7.45±0.200 | 138.4 | 0.45 | 12.50 |
| NiAlO_x_-600 | 63.01 | 10.35 | 8.65±0.118 | 115.6 | 0.35 | 11.80 |

[a] The contents of Ni and Al were determined by inductively coupled plasma-atomic emission spectroscopy (ICP-AES). [b] Ni particle size was achieved based on TEM results. [c] Obtained from Brunauer-Emmett-Teller calculation according to N_2_ adsorption. [d] Calculated from the BJH adsorption cumulative volume of pores between 1.7 and 300 nm diameter. [e] Calculated by the BJH method from the desorption isotherm linear plot.

**Table S2.** Catalytic performance of various catalysts on hydrogenolysis of benzyl phenyl ether.^[a]^

| **Entry** | **Catalyst** | **Conversion (%)^[b]^** | **Yield (%)^[b]^** | | | |
| --- | --- | --- | --- | --- | --- | --- |
|  |  |  | **A** | **B** | **C** | **D** |
| 1 | MgAlO_x_-400 | 0 | 0 | 0 | 0 | 0 |
| 2 | CoAlO_x_-400 | 0 | 0 | 0 | 0 | 0 |
| 3 | CuAlO_x_-400 | 0 | 0 | 0 | 0 | 0 |
| 4 | NiAlO_x_-400 | >99.9 | 48.8 | 0 | 51.1 | 0 |

[a] Reaction conditions: Benzyl phenyl ether, 1.0 mmol; ethanol, 5 g; reaction temperature, 30 °C; reaction time, 4 h; 1 MPa H_2_; amount of catalyst, 45 mg. [b] Conversion and yield were determined by GC using *n*-dodecane as the internal standard.

**Table S3.** The lignin model compounds with different C-O linkages and their C-O bond dissociation energies.

| **Sample** | **Base sites (mmol/g)** | | | | **Acid sites (mmol/g)** | | | |
| --- | --- | --- | --- | --- | --- | --- | --- | --- |
|  | **B_CO2_^[a]^** | **B_W_** | **B_M_** | **B_S_** | **A_NH3_^[b]^** | **A_W_** | **A_M_** | **A_S_** |
| NiAlO_x_-300 | 1.11 | 0.59 | 0.52 | - | 1.29 | 0.21 | 1.08 | - |
| NiAlO_x_-400 | 1.16 | 0.55 | 0.49 | 0.004 | 1.11 | 0.23 | 0.84 | 0.12 |
| NiAlO_x_-500 | 0.94 | 0.44 | 0.43 | 0.56 | 0.88 | 0.22 | 0.66 | 0.07 |
| NiAlO_x_-600 | 0.86 | 0.36 | 0.32 | 0.38 | 0.83 | 0.21 | 0.62 | 0.18 |
| NiAlO_x_-300^[c]^ | 1.13 | 0.57 | 0.56 | - | 1.23 | 0.26 | 0.97 | - |

[a] B_CO2_ represented the total concentration of base sites, which was calculated based on the results of CO_2_-TPD. [b] A_NH3_ denoted the total concentration of acid sites, which was calculated based on the result of NH_3_-TPD. [c] The recovered NiAlO_x_-300 after four catalytic cycles.

**Table S4. Comparison of hydrogenation of benzyl phenyl ether over different catalysts.**

|  | | | | | |
| --- | --- | --- | --- | --- | --- |
| **Entry** | **Catalyst** | **Experimental conditions** | **Solvent** | **Conversion (%)** | **Ref** |
| 1 | Ru_15_Ni_85_NC | 95 °C, 1 bar H_2_ | Water | 99 | 2 |
| 2 | Pd/AC | 25 °C, 10 bar H_2_ | Methanol | 100 | 3 |
| 3 | Ru/4-1AC | 150 °C, 10 bar H_2_ | 2-Propanol | 100 | 4 |
| 4 | Ru/AC | 150 °C,10 bar H_2_ | Methanol | 88.8 | 5 |
| 5 | 10%Ni/α-Al_2_O_3_ | 95 °C, 5 bar H_2_ | 2-Propanol | 100 | 6 |
| 6 | Co/C@N | 160 °C, 20 bar H_2_ | n-Hexane | 100 | 7 |
| 7 | Ni/CeO_2_ | 120 °C, 20 bar H_2_ | n-Hexane | 100 | 8 |
| 8 | 1.5% Ni/CeO_2_ | 200 °C, 50 bar H_2_ | Water | 80 | 9 |
| 9 | Ni/AlP_0.5_O_x_ | 30 °C, 30 bar H_2_ | Methanol | 100 | 10 |
| 10 | NiAlO_x_ | 20 °C, 10 bar H_2_ | Ethanol | 100 | This work |

**Table S5.** Effect of solvents on hydrogenolysis of benzyl phenyl ether.^[a]^

| **Entry** | **Solvent** | **Con. (%)^[b]^** | **Yield (%)^[b]^** | | | |
| --- | --- | --- | --- | --- | --- | --- |
|  |  |  |  |
| 1 | Isopropanol | >99.99 | 49.7 | trace | 50.2 | trace |
| 2 | Methanol | >99.99 | 47.9 | trace | 52.1 | trace |
| 3 | Ethanol | >99.99 | 48.8 | trace | 51.1 | trace |
| 4 | Propanol | >99.99 | 50.1 | trace | 49.8 | trace |
| 5 | 1-butanol | 96.30 | 46.3 | trace | 50.0 | trace |
| 6 | n-hexane | 27.67 | 14.5 | trace | 13.1 | trace |
| 7 | water | 0 | 0 | 0 | 0 | 0 |
| 8 | n-hexane (90%) + Ethanol (10%) | >99.99 | 49.2 | trace | 50.4 | trace |

[a] Reaction condition: Benzyl phenyl ether, 1.0 mmol; solvent, 5 g; reaction temperature, 30 °C; reaction time, 4 h; 1 MPa H_2_; amount of catalyst, 45 mg. [b] Conversion and yield were determined by GC using n-dodecane.

**Table S6.** The lignin model compounds with different C-O linkages and their C-O bond dissociation energies.^[2]^

| **Entry** | **Substrate** | **BDE (kJ/mol)** |
| --- | --- | --- |
| 1 |  | 245 |
| 2 |  | 296 |
| 3 |  | 314 |

**Table S7.** Ni dispersion of the fabricated NiAlO_x_-T materials.^[a]^

| **Sample** | **Metal dispersion (%)** | **surface Ni^0^ concentration (mmol·g^-1^)** |
| --- | --- | --- |
| NiAlO_x_-300 | 12.07 | 1.218 |
| NiAlO_x_-400 | 10.22 | 1.064 |
| NiAlO_x_-500 | 7.78 | 0.791 |
| NiAlO_x_-600 | 6.34 | 0.658 |

[a] Surface Ni dispersion is calculated based on the results of H_2_ pulse chemisorption at 333 K.

**References**

1. S. Liu, M. Dong, Y. Wu, S. Luan, Y. Xin, J. Du, S. Li, H. Liu, B. Han, “Solid surface frustrated Lewis pair constructed on layered AlOOH for hydrogenation reaction”, *Nat. Commun.* **2022**, *13*, 2320.
2. S. Bulut, S. Siankevich, A. P. van Muyden, D. T. L. Alexander, G. Savoglidis, J. G. Zhang, V. Hatzimanikatis, N. Yan, P. J. Dyson, “Efficient cleavage of aryl ether C-O linkages by Rh-Ni and Ru-Ni nanoscale catalysts operating in water”, *Chem. Sci.* **2018**, *9*, 5530-5535.
3. T. Xie, J.-P. Cao, C. Zhu, X.-Y. Zhao, M. Zhao, Y.-P. Zhao, X.-Y. Wei, “Selective cleavage of C-O bond in benzyl phenyl ether over Pd/AC at room temperature”, *Fuel Processing Technology.* **2019**, *188*, 190-196.
4. W. Jiang, J.-P. Cao, C. Zhu, J.-X. Xie, L. Zhao, C. Zhang, X.-Y. Zhao, Y.-P. Zhao, H.-C. Bai, “Selective hydrogenolysis of C-O bonds in lignin and its model compounds over a high-performance Ru/AC catalyst under mild conditions”, *Chem. Eng. Sci.* **2022**, *253*, 117554.
5. J.-P. Cao, T. Xie, X.-Y. Zhao, C. Zhu, W. Jiang, M. Zhao, Y. P. Zhao, X.-Y. Wei, “Selective cleavage of ether C-O bond in lignin-derived compounds over Ru system under different H-sources”, *Fuel.* **2021**, *284*, 119027.
6. W. Jiang, J.-P. Cao, Z.-X. Huan, X. Hu, W. Tang, C.-X. Chen, H.-Y. Wang, Z.-M. He, X.-Y. Zhao, H.-C. Bai, “Relatively Electron-Rich Ni Nanoparticles Supported on α-Al_2_O_3_ for High-Efficiency Hydrogenolysis of Lignin and Its Derivatives under Mild Conditions”, *ACS Sustainable Chem. Eng.* **2023**, *11*, 17646-17661.
7. Q.-L. Song, Y.-P. Zhao, F.-P. Wu, G.-S. Li, X. Fan, R.-Y. Wang, J.-P. Cao, X.-Y. Wei, “Selective hydrogenolysis of lignin-derived aryl ethers over Co/C@N catalysts”, *Renew. Energ.* **2020**, *148*, 729-738.
8. J. Zhao, X.-Y. Wei, J.-H. Lv, Q.-Q. Kong, J.-H. Li, Z. Li, Z.-C. Fan, L. Li, Y.-Y. Zhang, Z.-M. Zong, “Catalytic hydroconversion of two lignin-related model compounds over Ni/CeO_2_”, *Fuel.* **2023**, *331*, 125808.
9. J. Xie, Y. Xi, W. Gao, H. Zhang, Y. Wu, R. Zhang, H. Yang, Y. Peng, F. Li, Z. Li, C. Li, “Hydrogenolysis of lignin model compounds on Ni nanoparticles surrounding the oxygen vacancy of CeO_2_”, *ACS Catal.* **2023**, 13, 9577-9587.
10. L. Jiang, G. Xu, Y. Fu, “Catalytic cleavage of the C-O bond in lignin and lignin-derived aryl ethers over Ni/AlP_y_O_x_ catalysts”, *ACS Catal.* **2022**, *12*, 9473-9485.
